# MobileNetV2 for Multiclass Neurodegenerative Gait Analysis Using Bilateral Ground Reaction Force Images: From Transfer Learning to Raspberry Pi 4 Deployment

**DOI:** 10.3390/s26217010

**Published:** 2026-11-05

**Authors:** Vessela Krasteva, Todor Stoyanov, Irena Jekova

**Affiliations:** Institute of Biophysics and Biomedical Engineering, Bulgarian Academy of Sciences, Acad. G. Bonchev Str. Bl. 105, 1113 Sofia, Bulgaria; vessika@biomed.bas.bg (V.K.); todor@biomed.bas.bg (T.S.)

**Keywords:** gait classification, neurodegenerative diseases, vertical ground reaction force, amyotrophic lateral sclerosis, Huntington’s disease, Parkinson’s disease, image processing, deep learning, edge computing, embedded systems

## Abstract

Neurodegenerative diseases (NDDs), including Parkinson’s disease (PARK), Huntington’s disease (HUNT), and amyotrophic lateral sclerosis (ALS), are associated with characteristic gait alterations. This study proposes an image-based deep-learning approach for NDD gait classification and its implementation on an edge-computing platform. The main contributions include: (1) mapping synchronized left- and right-foot ground reaction force (GRF) signals from individual gait cycles into a common 2D bilateral force space to generate three complementary representations—Trajectory Map (bilateral force trajectories), Derivative Map (dynamic transitions), and Occupancy Heatmap (loading-state distributions); (2) adapting ImageNet-pretrained MobileNetV2 to four-class gait classification (CONTROL, ALS, HUNT, and PARK) through classification-layer retraining and feature-extractor fine-tuning; (3) comparing compact (α = 0.35; ~0.4 M parameters) and higher-capacity (α = 1.4; ~4.4 M parameters) MobileNetV2 configurations; and (4) evaluating the deployment of the resulting models on a Raspberry Pi 4 as a resource-constrained edge-computing platform. The models were evaluated on a test subset of 2259 gait cycles from the Gait in Neurodegenerative Disease Database, comprising CONTROL (612), ALS (367), HUNT (727), and PARK (553). Fine-tuning increased overall four-class accuracy from 89.64–92.61% to 95.09–96.41% across the GRF representations and model configurations. Paired comparisons showed improvements of 3.81–6.29 percentage points across all six matched configurations. Class-specific one-vs-rest accuracy was consistently high after fine-tuning: CONTROL (97.74–98.63%), ALS (98.41–98.94%), HUNT (96.02–97.21%), and PARK (97.30–98.23%). The highest four-class accuracy (96.41%; 95% CI: 95.45–97.29%; Occupancy Heatmap, α = 1.4) was preserved for the unquantized TFLite model evaluated on the Raspberry Pi 4, while dynamic-range quantization reduced accuracy by 0.22 percentage points to 96.19%. Compact fine-tuned MobileNetV2 models required approximately 42–46 ms of inference latency and 89–97 mJ of estimated incremental energy per inference, compared with 324–374 ms and 680–785 mJ, respectively, for the higher-capacity models. Dynamic-range quantization reduced model storage by approximately 62% (α = 0.35) and 72% (α = 1.4). Overall, bilateral GRF representations combined with MobileNetV2 transfer learning enable accurate multiclass NDD gait classification, while compact models provide a favorable performance–efficiency trade-off for resource-constrained edge implementation. However, subject-independent validation remains necessary because the reported accuracy was obtained using gait-cycle-level data partitioning.

## 1. Introduction

Neurodegenerative diseases (NDDs) are among the leading causes of disability and mortality worldwide [1]. Among them, Parkinson’s disease (PARK) is associated with the progressive degeneration of dopamine-producing neurons, Huntington’s disease (HUNT) is caused by a genetic mutation in the huntingtin gene, and amyotrophic lateral sclerosis (ALS) is characterized by the progressive degeneration of nerve cells in the brain and spinal cord [2,3,4,5,6,7]. These diseases are associated with distinct gait abnormalities, including:

- PARK: reduced gait velocity and stride length, increased cadence, shuffling steps with insufficient foot clearance, episodic freezing of gait, and a tendency toward rapid, short, involuntary steps in the forward or backward direction [8,9,10,11,12];
- HUNT: an unsteady gait characterized by alternating hyperkinesia and hypokinesia, reduced gait velocity, stride length, and cadence, and an increased stance phase [7,13,14];
- ALS: reduced gait velocity, cadence, and stride length, decreased single-leg support time and increased double-leg support time during walking, and greater gait variability compared with healthy subjects, particularly in stance and swing times [15,16,17].

Movement disturbances are among the early symptoms of PARK, HUNT, and ALS, making the analysis of gait dynamics and associated abnormalities a potentially valuable approach for their assessment and differentiation [18,19,20,21]. In this context, features derived from vertical ground reaction force (GRF) signals have been widely investigated, including temporal gait parameters related to swing, stance, and double-support intervals [22,23,24,25,26]; statistical, entropy-based, and nonlinear features extracted from gait time series [25,27,28,29,30,31,32,33,34]; correlation-based features [35,36]; spectral and cepstral features [25,27,29]; features obtained using detrended fluctuation analysis and dynamic time warping [35], recurrence quantification analysis [30]; and topological features derived from persistence landscapes of the gait time series [37]. These handcrafted features have subsequently been used for NDD classification with a broad range of machine-learning approaches, including decision trees [22,25,28,33,37], K-nearest neighbors (KNN) [22,25,27,31,33,35,37], support vector machines (SVM) [25,26,27,28,31,33,34,35], linear regression (LR) [28], multiclass logistic regression (MLR) [25], linear discriminant analysis (LDA) [28], gradient boosting (GB) [28], Naive Bayes (NB) [37], Adaptive Neuro-Fuzzy Inference Systems (ANFIS) [23], and neural networks (NNs) [22,24,27,29,30,32,33,35,36]. More recently, approaches that operate directly on raw gait signals have also been investigated, including bidirectional long short-term memory (BiLSTM) networks [38] and deep-learning architectures combining dilated causal convolutions for local gait-pattern extraction with multi-head self-attention for modeling long-range dependencies [39].

In recent years, different gait data modalities have been transformed into two-dimensional (2D) image representations to enable their analysis using convolutional neural networks (CNNs). These approaches differ in the gait information encoded and the transformation used to generate the images. Erdaş and Sümer [40] represented handcrafted temporal gait features, including swing, stance, and double-support intervals, as QR-code images. Other studies transformed vertical GRF signals into time–frequency spectrograms [38,41], thereby representing the evolution of their spectral characteristics. Torghabeh et al. [42] investigated the interaction between left- and right-foot GRF signals using wavelet coherence analysis and color-coded images, explicitly incorporating bilateral relationships in the time–frequency domain. Li et al. [43] constructed images representing left- and right-foot stance, swing, and stride intervals together with double-support intervals, whereas Lin et al. [44] employed recurrence plots to characterize repeated patterns in GRF time series. Thus, existing image-based approaches primarily encode handcrafted temporal features, time–frequency characteristics, interlimb coherence, gait-phase intervals, or recurrence relationships.

Beyond the differences in image-generation methods, previous studies have also adopted different approaches to feature extraction and classification. Some studies have employed specifically designed classification architectures, including a CNN [40] and a CNN–BiLSTM model [43], whereas others have used CNN architectures pretrained on the ImageNet dataset [45], including AlexNet [38,41,42,44], GoogLeNet [38,42], VGG16 [38], and SqueezeNet [42]. Depending on the study, these pretrained architectures have been used for feature extraction or adapted for classification, allowing learned image features to be incorporated into the diagnostic decision process. Pretrained networks and transfer learning are particularly attractive for gait classification, as they can reduce the dependence on large task-specific datasets by exploiting visual features learned from large-scale image databases [46,47].

Most of the studies discussed above address binary classification, primarily distinguishing individual NDDs from healthy controls (ALS vs. CONTROL, PARK vs. CONTROL, or HUNT vs. CONTROL) [22,23,24,25,26,28,29,30,31,32,33,34,35,37,41,43,44]. Some studies additionally investigate pairwise discrimination among NDDs (ALS vs. PARK, ALS vs. HUNT, or PARK vs. HUNT) (ALS vs. PARK, ALS vs. HUNT, PARK vs. HUNT) [25,26,30,31,33,34,41,44], while PARK has also been distinguished from all other groups as a binary classification task [27]. In contrast, fewer studies have addressed the more challenging multiclass problem of simultaneously discriminating among ALS, PARK, HUNT, and CONTROL subjects [22,25,31,35,36,38,39,40,41,42,44], highlighting the comparatively limited investigation of multiclass NDD classification.

Beyond classification performance, the implementation of gait-analysis algorithms on resource-constrained computing platforms is important for the development of portable systems capable of performing local data processing and inference. On-device inference can reduce latency and dependence on continuous network connectivity associated with cloud-based processing. Accordingly, recent studies have investigated the implementation of gait-analysis algorithms on embedded computing platforms with limited computational resources. Most of these implementations have focused on the detection of freezing-of-gait (FOG) events in PARK patients [48,49,50,51,52], assessment of functional recovery in PARK patients [53], and gait estimation [54,55], primarily through the analysis of acceleration and/or angular-velocity signals [48,49,50,51,52,53,54,55]. The corresponding algorithms have been implemented on conventional microcontrollers [48,49,50,53] or Raspberry Pi single-board computers [51,52,54,55]. However, embedded implementation of multiclass NDD classification remains largely unexplored. To the best of our knowledge, only one study has reported multiclass discrimination among ALS, PARK, HUNT, and CONTROL subjects using an FPGA-implemented decision-tree classifier based on stride time, stride-time variability, and an autocorrelation decay factor [56].

The aim of this study is to investigate deep-learning classification of CONTROL, ALS, HUNT, and PARK gait cycles using novel 2D images derived from synchronized bilateral GRF signals. The images characterize three aspects of bilateral gait dynamics: the GRF trajectory, dynamic force transitions, and occupancy of bilateral GRF states. This formulation produces fixed-size images independent of gait-cycle duration, avoiding temporal resampling for image-based deep-learning models. Their classification potential is evaluated through transfer learning using MobileNetV2, a lightweight CNN pretrained on ImageNet, comparing compact (α = 0.35) and higher-capacity (α = 1.4) configurations that differ approximately 10.5-fold in model parameters. Transfer learning is performed through classification-layer retraining and feature-extractor fine-tuning. Finally, the resulting models are evaluated on a Raspberry Pi 4 to investigate the feasibility of resource-constrained edge implementation in terms of classification performance and computational requirements. The findings may support the future development of portable, on-device systems for NDD gait classification.

## 2. Materials and Methods

### 2.1. Gait Database

The present study used data from the Gait in Neurodegenerative Disease Database (GNDD, Version 1.0.0) [16,57,58], publicly available via the PhysioNet archive. The database contains 64 gait recordings, including 16 healthy controls (CONTROL) and 48 patients distributed across three NDD groups: 20 with HUNT, 15 with PARK, and 13 with ALS. The demographic characteristics of the four groups are summarized in Table 1. Data were fully anonymized prior to public release, and only non-identifiable subject codes are available to secondary users. The original data collection was conducted under institutional ethics committee approval, and secondary research use of the dataset is permitted under the PhysioNet data-sharing agreement.

Raw gait data were acquired using force-sensitive resistors placed under each foot, with sensor output approximately proportional to the GRF. For each subject, the database provides two binary files (.let and .rit) containing synchronized left- and right-foot GRF signals recorded during approximately 5 min of walking, together with a header file (.hea) and a derived time-series file (.ts) containing stride-level gait parameters. The GRF signals were sampled at 300 Hz. The initial 20 s of each recording were excluded to remove the walking-initiation phase and retain steady-state gait. For subject ALS5, data recorded after 168 s were additionally excluded because of sensor failure and subsequent signal loss. Across the dataset, the number of available gait cycles per recording ranged from 122 to 310, with a mean of 237 ± 35. Their distribution across the four groups is summarized in Table 1.

### 2.2. Two-Dimensional Bilateral GRF Representations of the Gait Cycle

The elapsed-time values provided in the .ts file were used as temporal markers delimiting successive gait cycles and for extracting the corresponding synchronized left- and right-foot GRF signal segments from the original recordings. Each gait cycle is therefore represented by two synchronized one-dimensional GRF signals.

In this study, the one-dimensional GRF signals corresponding to a complete gait cycle were transformed into a 2D bilateral GRF representation and subsequently rendered as an RGB image. In the bilateral representation, the left- and right-foot GRF signals define a common bilateral force space, where the left-foot signal is assigned to the horizontal axis and the right-foot signal to the vertical axis. Consequently, each synchronized sample within a gait cycle is represented by the point:

p(n) = (F_L_(n), F_R_(n)), n = 1,…,N, (1)

where F_L_(n) and F_R_(n) denote the synchronized left- and right-foot GRF signals at sample n, and N is the number of samples in the gait cycle.

Unlike conventional one-dimensional time-series representations, in which the signal length depends directly on gait-cycle duration and often requires temporal normalization to a fixed number of samples, the proposed bilateral GRF representations are constructed in a bilateral force space rather than a time domain. Consequently, gait cycles of different durations are mapped to images of identical spatial dimensions, while temporal information is preserved through the sequence of synchronized bilateral force coordinates. Furthermore, because the bilateral GRF trajectory forms a closed loop in the bilateral force space, the resulting image represents the complete gait cycle as a whole and is not dependent on alignment to a specific fiducial event, such as heel strike or toe-off.

Three bilateral GRF representations were investigated, all sharing the same bilateral force-space coordinates defined by Equation (1). They differ in the quantity encoded at these coordinates, thereby emphasizing different characteristics of the gait cycle:

1. Bilateral GRF Trajectory Map, representing the geometry and temporal progression of the bilateral GRF trajectory throughout the gait cycle.
2. Bilateral GRF Derivative Map, representing the local rate and direction of GRF change within the bilateral force space.
3. Bilateral GRF Occupancy Heatmap, representing the spatial distribution of bilateral GRF states according to their frequency of occurrence during the gait cycle.

**Figure 1:**
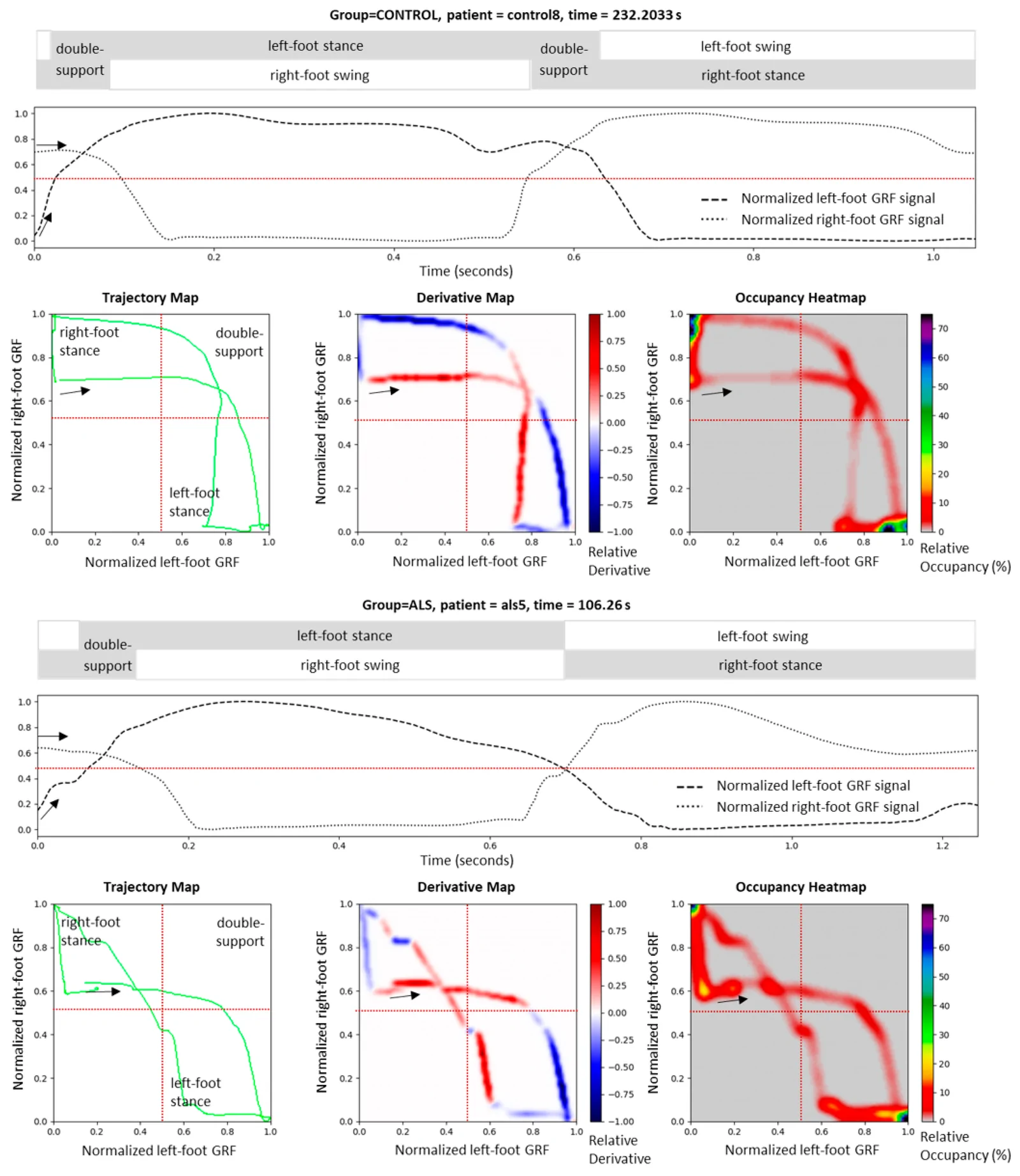

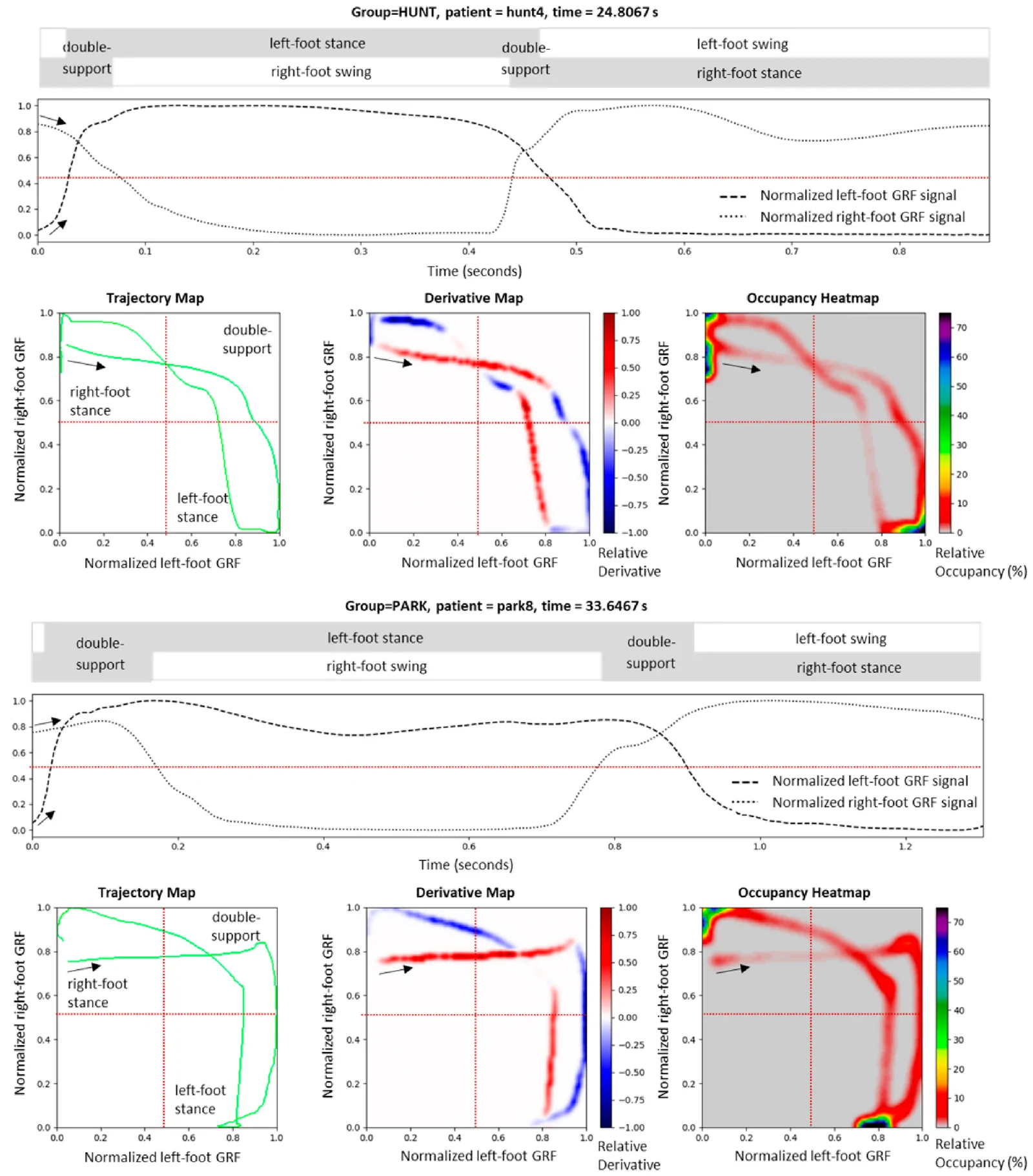
Representative gait cycles from the four diagnostic groups: healthy controls (CONTROL), amyotrophic lateral sclerosis (ALS), Huntington’s disease (HUNT), and Parkinson’s disease (PARK). For each example, the normalized left- and right-foot ground reaction force (GRF) signals are shown above the corresponding bilateral GRF Trajectory Map, Derivative Map, and Occupancy Heatmap. A normalized GRF threshold of 0.5 is used to distinguish stance and swing phases, including periods of double support. Red dotted lines indicate the threshold in the time-series plots and the corresponding thresholds in the bilateral GRF representations. Arrows indicate the temporal progression of the gait cycle. Patient identifiers and gait-cycle extraction times are provided for the illustrated examples.

The characteristics of the three representations are summarized in Table 2. Figure 1 is provided to illustrate the three bilateral GRF representations derived from the raw GRF signals for representative gait cycles from the different diagnostic groups. The construction and interpretation of these representations are described in the following sections.

#### 2.2.1. Preprocessing of GRF Signals

Prior to generating the bilateral GRF representations, each extracted gait cycle was examined for missing (NaN) samples. When present, missing samples were replaced using forward filling by propagating the nearest preceding valid sample. Leading missing samples, which cannot be forward-filled, were replaced with the first available measurement.

The left- and right-foot GRF signals were then independently normalized to the interval [0, 1] using min–max normalization:

(2) $$\hat{F_{q}}(n)=\frac{F_{q}(n)-\min(F_{q})}{\max\left(F_{q}\right)-\min(F_{q})},q\in \{L,R\},$$

where F_q_(n) and $\hat{F_{q}}(n)$ denote the original and normalized GRF signals, respectively. Examples of the normalized left- and right-foot GRF signals are shown in Figure 1 (top traces) for representative gait cycles from the four diagnostic groups. For illustrative purposes, the corresponding gait phases (left and right stance and swing phases, together with periods of double support) were determined using a normalized GRF threshold of 0.5 and are also indicated in the figure.

After normalization, the bilateral force-space coordinates become:

(3) $$\hat{p}\left(n\right)=\left({\hat{F}}_{L}\left(n\right),{\hat{F}}_{R}\left(n\right)\right),$$

which define the spatial location of each synchronized GRF sample within the normalized bilateral force space [0, 1] × [0, 1] and serve as the common basis for constructing all three bilateral GRF representations. Unlike conventional time-series representations, the horizontal and vertical axes correspond exclusively to the normalized left- and right-foot GRF signals rather than to elapsed time. Temporal information is preserved through the ordered sequence of synchronized bilateral force coordinates. Although all three representations share the same bilateral force-space coordinates, they differ in the quantity encoded at each coordinate, thereby emphasizing complementary characteristics of the gait cycle. Representative examples of the resulting Trajectory Map, Derivative Map, and Occupancy Heatmap are shown in Figure 1, while the construction of each representation is described in the following sections.

#### 2.2.2. Bilateral GRF Trajectory Map

The Bilateral GRF Trajectory Map (Trajectory Map) represents the temporal evolution of the synchronized bilateral GRF pattern throughout the gait cycle. The normalized bilateral coordinates defined in Equation (3) determine the spatial location of each synchronized GRF sample within the bilateral force space. Consecutive samples were connected in temporal order to form a continuous trajectory. The bilateral force space was discretized into a regular 100 × 100 grid spanning [0, 1] × [0, 1]. The trajectory was rasterized onto the grid by connecting consecutive bilateral force coordinates with straight line segments. The resulting trajectory map was smoothed using a Gaussian filter to obtain a continuous representation. Since the Trajectory Map represents only the geometric evolution of the bilateral GRF pattern without encoding an additional scalar quantity, the trajectory was represented using only the green channel of the RGB image, while the remaining channels were set to zero. Representative Trajectory Maps are shown in Figure 1, illustrating how the normalized left- and right-foot GRF signals are transformed into continuous trajectories.

#### 2.2.3. Bilateral GRF Derivative Map

The Bilateral GRF Derivative Map (Derivative Map) represents the local rate of change of the synchronized bilateral GRF signals within the bilateral force space. The first derivatives of the normalized left- and right-foot GRF signals were calculated from the differences between consecutive samples, n and n − 1, using the original sampling interval:

(4) $$d\hat{F_{q}}\left(n\right)=\frac{{\hat{F}}_{q}\left(n\right)-{\hat{F}}_{q}\left(n-1\right)}{\Delta t},q\in \left\{L,R\right\},n=1,\ldots ,N-1,$$

where Δt = 1/f*_s_* and f_s_ = 300 Hz.

The bilateral GRF derivative was calculated as the sum of the left- and right-foot GRF derivatives:

(5) $$d\hat{F}\left(n\right)=d{\hat{F}}_{L}\left(n\right)+d{\hat{F}}_{R}\left(n\right),\;n=1,\ldots ,N-1.$$

The normalized bilateral coordinates defined in Equation (3) determine the spatial location of each sample within the bilateral force space, whereas the corresponding bilateral derivative $d\hat{F}\left(n\right)$ defined in Equation (5) determines the pixel value assigned to the corresponding bilateral force coordinates. The bilateral force space was discretized into a regular 100 × 100 grid spanning [0, 1] × [0, 1], with all grid cells initially assigned a value of zero. The bilateral derivative values were then mapped to the corresponding grid cells according to their bilateral force coordinates. When multiple samples were assigned to the same grid cell, their derivative values were averaged, yielding the mean bilateral GRF derivative represented by that cell. The resulting derivative map was smoothed using a Gaussian filter to obtain a continuous representation. For visualization, the smoothed bilateral derivative values were linearly normalized to the range [−1, 1], yielding a relative bilateral derivative while preserving the sign of the derivative. Finally, the relative bilateral derivative values were visualized using a diverging blue–white–red colormap centered at zero, where blue and red represent negative and positive bilateral derivatives, respectively, and white represents values close to zero.

Representative Derivative Maps are shown in Figure 1, illustrating how the bilateral GRF derivatives are encoded along the same bilateral trajectory as the Trajectory Map.

#### 2.2.4. Bilateral GRF Occupancy Heatmap

The Bilateral GRF Occupancy Heatmap (Occupancy Heatmap) represents the frequency of occurrence of synchronized bilateral GRF states within the bilateral force space. The occupancy of each grid cell was calculated as the number of synchronized GRF samples mapped to that cell:

(6) $$H\left(i,j\right)=\sum_{n=1}^{N}I_{i,j}\left(n\right),$$

where H(i,j) denotes the occupancy value of the (i, j)-th grid cell; i and j denote the horizontal and vertical grid indices, respectively, with i, j ∈ {1, …, 100}; and I*_i_*_,*j*_(*n*) is an indicator function. Letting C_i,j_ denote the corresponding grid cell in the bilateral force space, the indicator function is defined as:

(7) $$I_{i,j}\left(n\right)=\{\begin{array}{c} 1,\mathrm{if}\left({\hat{F}}_{L}\left(n\right),{\hat{F}}_{R}\left(n\right)\right)\in C_{i,j},\\ 0,\mathrm{otherwise}. \end{array}$$

The normalized bilateral coordinates defined in Equation (3) determine the spatial location of each synchronized GRF sample within the bilateral force space, whereas the corresponding occupancy value H(i, j) determines the value assigned to each grid cell. The bilateral force space was discretized into a regular 100 × 100 grid spanning [0, 1] × [0, 1], with all grid cells initially assigned a value of zero. The occupancy counts were obtained by mapping the bilateral force coordinates to their corresponding grid cells. The resulting occupancy map was smoothed using a Gaussian filter and subsequently normalized to the range 0–100 by dividing all occupancy values by the maximum value of the smoothed occupancy map and multiplying by 100. Finally, the normalized occupancy values were visualized using the reversed NIPY Spectral colormap, with color indicating the relative occupancy magnitude, ranging from gray for zero or low occupancy to blue/green for the highest occupancy. The resulting image was then resized to 224 × 224 pixels.

Representative Occupancy Heatmaps are shown in Figure 1, illustrating the frequency with which different regions of the bilateral GRF trajectory are occupied during the gait cycle.

#### 2.2.5. Interpretation of the Bilateral GRF Representations

The bilateral GRF representation changes the description of gait from force evolution over time to the relationship between the GRFs of the two feet. Figure 1 illustrates these relationships for representative gait cycles from CONTROL, ALS, HUNT, and PARK, showing visually distinct bilateral patterns.

In the Trajectory Map (Figure 1, bottom-left panels), the green curve forms a bilateral trajectory through successive loading states. Relatively stable unilateral loading forms characteristic loops in the upper-left quadrant (right-foot stance) and lower-right quadrant (left-foot stance), while changes in relative bilateral loading connect these loops. Consequently, short temporal GRF transitions become spatially extended and more clearly visible than in the time domain, particularly during double support, when the trajectory passes through the upper-right bilateral-loading quadrant. The CONTROL example shows relatively similar right- and left-foot stance loops connected through this quadrant. In comparison, the ALS example exhibits a more irregular and asymmetric trajectory, with a smaller and differently shaped right-foot-stance loop and an altered transition toward the opposite left-foot-stance state. The HUNT example shows a smoother trajectory than ALS, although its right-foot-stance loop is also less developed than that of the CONTROL example. In the PARK example, both stance-related loops are well represented but differ markedly in shape, with a distinctive transition through the double-support quadrant. These examples illustrate how the geometry of the bilateral trajectory and its right- and left-foot stance loops can reveal differences in interlimb force coordination that are less apparent in the time-domain GRF signals.

In the Derivative Map (Figure 1, bottom-middle panels), the trajectory geometry is complemented by information on the local rate and direction of GRF change. Red indicates a positive bilateral derivative, blue indicates a negative bilateral derivative, and white corresponds to derivative values close to zero. Following the trajectory from the arrow, the colored loops reflect the sequential loading and unloading of the two feet. Together with their position in the bilateral force space, the characteristic red–blue–red–blue progression can be interpreted as four successive phases of force change: (1) right-foot loading (positive derivative), (2) right-foot unloading (negative derivative), (3) left-foot loading (positive derivative), and (4) left-foot unloading (negative derivative). Thus, positive derivatives characterize force increase during loading in stance, whereas negative derivatives characterize force reduction during unloading toward swing. The CONTROL example shows relatively balanced derivative intensities for the two feet, including clear transitions toward near-zero values within the double-support region. In comparison, the ALS example shows unequal unloading patterns, with a more intense blue color for the right-foot unloading compared to the left-foot unloading, indicating different rates of force reduction. The HUNT example also shows unequal unloading patterns, including an interruption of the left-foot negative-derivative region by near-zero values. In the PARK example, the unloading patterns of the two feet again differ, with the left-foot transition initially showing relatively small force changes before developing a stronger negative derivative. Thus, the Derivative Map adds information on the dynamics of bilateral force development and reduction that is not represented by trajectory geometry alone.

In the Occupancy Heatmap (Figure 1, bottom-right panels), the trajectory geometry is complemented by a color-coded occupancy map indicating how frequently different bilateral GRF states are occupied during the gait cycle. Higher color intensities represent greater accumulation of samples and, because the GRF signals are uniformly sampled, correspond to longer residence in particular bilateral loading states. In all four examples, the highest occupancies occur predominantly in the upper-left and lower-right quadrants, corresponding to right- and left-foot stance, respectively, with high GRF in one foot and low GRF in the opposite foot. The connecting trajectory through the double-support quadrant shows lower occupancy, consistent with the shorter duration of this transition. This is particularly evident in the CONTROL example, where pronounced occupancy is observed in both right- and left-foot stance regions, while the transition through double support has comparatively low occupancy. The ALS example shows a broader high-occupancy region associated with right-foot stance, whereas the occupancy associated with left-foot stance is more dispersed along the lower-right loop. The HUNT example also shows concentrated occupancy in the right- and left-foot stance regions, but with a different distribution along the right-foot stance loop and its transition toward double support. In the PARK example, occupancy is strongly concentrated around the left-foot stance region, while the distribution along the upper and right portions of the trajectory differs from that of the other examples. Thus, the Occupancy Heatmap shows that the bilateral trajectory is not occupied uniformly and adds information on the relative time spent in different GRF states that is not conveyed by trajectory geometry or force-change dynamics alone.

### 2.3. Deep Transfer Learning Using MobileNetV2

#### 2.3.1. MobileNetV2 Model Configuration

MobileNetV2 [59] is a lightweight CNN architecture specifically designed for computationally efficient image processing on mobile and embedded platforms. It is based on inverted residual blocks with linear bottlenecks and depthwise separable convolutions, which reduce computational complexity and the number of parameters while maintaining effective feature extraction [59]. These characteristics make MobileNetV2 particularly suitable for the present study, where both classification performance and subsequent deployment on a resource-constrained computing platform are considered.

The MobileNetV2 models were instantiated from the implementation provided in TensorFlow Keras Applications (TensorFlow version 2.9.1) and initialized with weights pretrained on ImageNet. RGB images with input dimensions of 224 × 224 × 3 pixels were used for both configurations. For transfer learning, the network can be considered as comprising a pretrained feature extractor and a task-specific classification layer. The feature extractor transforms the input RGB image through the convolutional layers of MobileNetV2 into a compact feature vector containing high-level image features used for classification. The original ImageNet classification layer was replaced by a fully connected Dense layer with four output units [Dense(4)], corresponding to the CONTROL, ALS, HUNT, and PARK classes, followed by a softmax activation that provides the corresponding class probabilities.

The computational complexity and representational capacity of MobileNetV2 can be adjusted through the width multiplier α, which controls the number of channels while preserving the overall network structure [59]. In the present study, two substantially different configurations were investigated: α = 0.35, representing a compact model, and α = 1.4, representing a higher-capacity model. The two configurations differ substantially in feature-vector dimensionality and parameter count, with 415,332 and 4,370,884 total parameters, respectively, as summarized in Table 3. Given their approximately tenfold difference in parameter count, these configurations enable the trade-off between classification performance and computational requirements to be investigated, particularly in view of subsequent on-device implementation.

#### 2.3.2. Deep Transfer Learning Strategy

**Figure 2:**
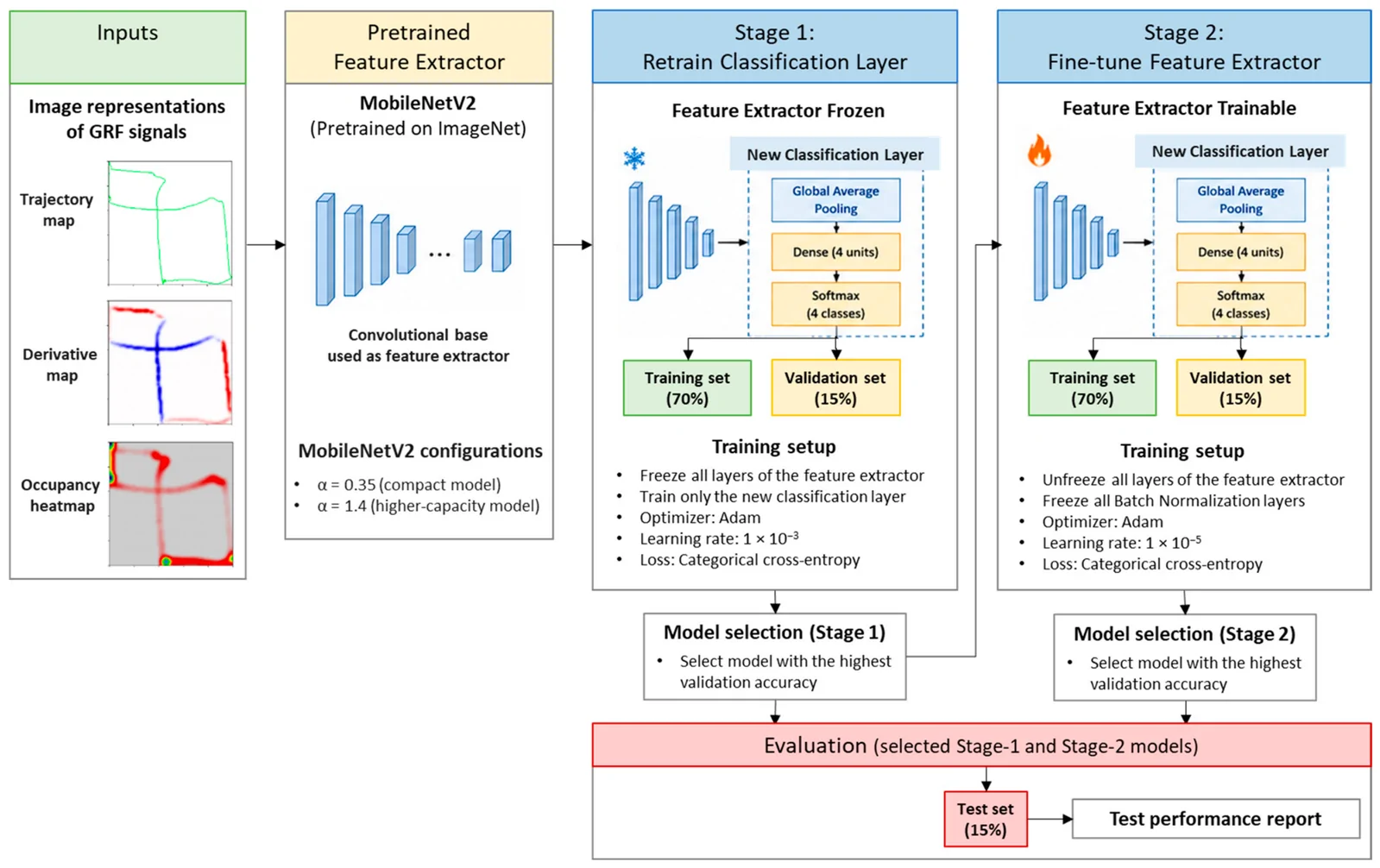
Two-stage transfer learning strategy for four-class gait-cycle classification using ImageNet-pretrained MobileNetV2 models. In the first stage, the pretrained feature extractor is frozen and only the newly introduced classification layer is trained. In the second stage, the feature extractor is fine-tuned using a lower learning rate, while the batch normalization layers remain frozen. The procedure is applied separately to the Trajectory Map, Derivative Map, and Occupancy Heatmap representations for both MobileNetV2 width multipliers (α = 0.35 and α = 1.4).

To adapt the ImageNet-pretrained MobileNetV2 models to the proposed gait classification task, a two-stage deep transfer learning strategy was employed, as illustrated in Figure 2. In the first stage, the pretrained feature extractor was kept frozen and only the newly introduced four-class classification layer was retrained. In the second stage, the feature extractor was unfrozen and fine-tuned using a substantially lower learning rate, while the Batch Normalization layers remained frozen. The same procedure was applied independently to each of the three bilateral GRF image representations and to both MobileNetV2 configurations (α = 0.35 and α = 1.4).

1. Stage 1: Retraining of the Classification Layer

During the first stage, all pretrained layers of the MobileNetV2 feature extractor were frozen and only the newly introduced four-class classification layer was trained. Consequently, only 5124 parameters for α = 0.35 and 7172 parameters for α = 1.4 were optimized (Table 3), substantially reducing the computational requirements of this initial training stage while preserving the ImageNet-pretrained feature-extraction parameters.

Model retraining was performed using the Adam optimizer with an initial learning rate of 10^−3^ and categorical cross-entropy as the loss function. A batch size of 128 was used, with training performed for a maximum of 1000 epochs. Early stopping was applied based on validation accuracy with a patience of 150 epochs, and the model achieving the highest validation accuracy was retained for subsequent fine-tuning.

2. Stage 2: Fine-tuning of the Feature Extractor

The model selected during Stage 1 was subsequently used to initialize the fine-tuning stage. The pretrained feature-extraction layers were unfrozen and optimized using a reduced learning rate of 10^−5^, allowing the pretrained feature representations to be further adapted to the bilateral GRF images.

The Batch Normalization layers remained frozen throughout the fine-tuning stage. These layers maintain normalization parameters and running estimates of feature means and variances learned during ImageNet pretraining. Updating them using the comparatively small gait dataset could alter the pretrained feature distributions and potentially reduce model stability and generalization [60]. Freezing these layers therefore preserves the pretrained normalization statistics while the remaining feature-extraction layers are adapted to the bilateral GRF representations. This resulted in 387,172 and 4,275,012 trainable parameters for the α = 0.35 and α = 1.4 configurations, respectively (Table 3).

Fine-tuning was performed using the same optimization protocol as in Stage 1, including the Adam optimizer, categorical cross-entropy loss, a batch size of 128, a maximum of 1000 epochs, and early stopping based on validation accuracy with a patience of 150 epochs. The model achieving the highest validation accuracy during fine-tuning was retained for subsequent performance evaluation.

#### 2.3.3. Performance Evaluation

##### Dataset Partitioning

The complete dataset comprised 15,056 gait cycles, which were randomly partitioned at the gait-cycle level into training (70%), validation (15%), and test (15%) subsets using stratified sampling according to diagnostic class and a fixed random state (42). The resulting subsets contained 10,539, 2258, and 2259 gait cycles, respectively. The distribution of gait cycles among the four diagnostic classes and dataset subsets is summarized in Table 4. The class proportions in the original dataset ranged from 16.3% for ALS to 32.2% for HUNT, indicating that no single diagnostic group dominated the dataset. Stratified partitioning preserved these relative class proportions across the training, validation, and test subsets. The training subset was used for model parameter optimization, whereas the validation subset was used for early stopping and model selection during both stages of the transfer learning procedure. The test subset was held out from model training and selection and was used exclusively for performance evaluation. Because partitioning was performed at the gait-cycle level rather than the participant level, gait cycles from the same participant could occur in different dataset subsets. Consequently, the reported test performance reflects classification of previously unseen gait cycles, but not necessarily generalization to previously unseen participants.

##### Classification Performance Metrics

Classification performance on the test subset was evaluated using precision, recall, specificity, F1-score, area under the receiver operating characteristic curve (AUC), one-vs-rest (OvR) accuracy, and overall four-class accuracy. Precision, recall, specificity, F1-score, AUC, and OvR accuracy were determined separately for each diagnostic class using an OvR approach, in which the considered class was treated as positive and the remaining three classes collectively as negative. Accordingly, true positives (TP), true negatives (TN), false positives (FP), and false negatives (FN) were determined separately for each class. The corresponding performance metrics were calculated as:

(8) $$\mathrm{Precision}=\frac{\mathrm{TP}}{\mathrm{TP}+\mathrm{FP}},\;\;\;\mathrm{Recall}=\frac{\mathrm{TP}}{\mathrm{TP}+\mathrm{FN}},\;\;\;\mathrm{Specificity}=\frac{\mathrm{TN}}{\mathrm{TN}+\mathrm{FP}},\;F1-\mathrm{score}=\frac{2\mathrm{TP}}{2\mathrm{TP}+\mathrm{FP}+\mathrm{FN}},\;\;\;\mathrm{Accuracy}\left(\mathrm{OvR}\right)=\frac{\mathrm{TP}+\mathrm{TN}}{\mathrm{TP}+\mathrm{TN}+\mathrm{FP}+\mathrm{FN}}$$

Receiver operating characteristic (ROC) curves were obtained separately for each diagnostic class according to the same OvR scheme using the predicted class probabilities, and the corresponding AUC was calculated as the area under each ROC curve. Precision, recall, specificity, F1-score, OvR accuracy, and four-class accuracy were expressed as percentages, whereas AUC was reported on a scale from 0 to 1.

In addition to the class-specific OvR metrics, overall four-class accuracy was calculated as the proportion of correctly classified gait cycles across all four diagnostic classes:

(9) $$\mathrm{Accuracy}\left(\mathrm{Four}-\mathrm{Class}\right)=\frac{\mathrm{Ncorrect}}{\mathrm{Ntotal}}\times 100,\%$$

where Ncorrect is the total number of correctly classified gait cycles and Ntotal is the total number of gait cycles in the test subset.

##### Statistical Analysis

To quantify uncertainty in classification performance and assess differences between MobileNetV2 configurations, subject-clustered bootstrap resampling was performed using 10,000 replicates and a fixed random seed (42). Subjects were sampled with replacement within each diagnostic class, retaining all their associated test gait cycles in each replicate. Overall four-class classification accuracy, as defined in Equation (9), was recalculated for each bootstrap sample, and percentile-based 95% confidence intervals were obtained from the resulting distributions.

Paired comparisons were performed using predictions from the same test gait cycles across model configurations. For each comparison, the difference in overall four-class accuracy was recalculated using the same resampled subjects for both models, and a 95% confidence interval was obtained from the bootstrap distribution of paired differences. A total of 24 paired comparisons were performed across three comparison families:

- GRF image representation comparisons: Twelve comparisons evaluated all three possible pairwise comparisons between the GRF image representations (Trajectory Map vs. Derivative Map, Trajectory Map vs. Occupancy Heatmap, and Derivative Map vs. Occupancy Heatmap), separately for each training strategy and network width multiplier.
- Network width comparisons: Six comparisons evaluated the two MobileNetV2 width multipliers (α = 0.35 vs. α = 1.4), separately for each GRF image representation and training strategy.
- Training-strategy comparisons: Six comparisons evaluated retrained versus fine-tuned models for each combination of GRF image representation and network width multiplier.

In each paired comparison, only one experimental factor was varied, while the remaining configuration factors were held constant.

Additionally, exact McNemar tests were applied to matched gait-cycle predictions, and the resulting *p*-values were adjusted using the Holm procedure separately within the three comparison families. A threshold of 0.05 was used for the adjusted *p*-values. Because multiple gait cycles originated from the same subjects, the McNemar tests did not account for within-subject dependence and were therefore considered exploratory. The subject-clustered bootstrap confidence intervals were used as the primary basis for interpreting differences between configurations. The confidence intervals were not adjusted for multiple comparisons.

##### Software and Hardware Environment

GRF signal processing, generation of the three bilateral GRF image representations, and implementation of the deep transfer learning procedure were performed in Python 3.9.23 using TensorFlow 2.9.1 and Keras. Model training and performance evaluation were conducted on a Persy Stinger workstation (PERSY Ltd. Sofia, Bulgaria) equipped with two Intel Xeon Silver 4214R processors operating at 2.4 GHz, 96 GB RAM, and an NVIDIA RTX A5000 GPU with 24 GB memory. Subsequent statistical analyses and visualization were performed in Python 3.13.14 using NumPy 2.4.6, pandas 3.0.5, SciPy 1.18.0, and Matplotlib 3.11.1.

### 2.4. MobileNetV2 Deployment on Raspberry Pi 4

**Figure 3:**
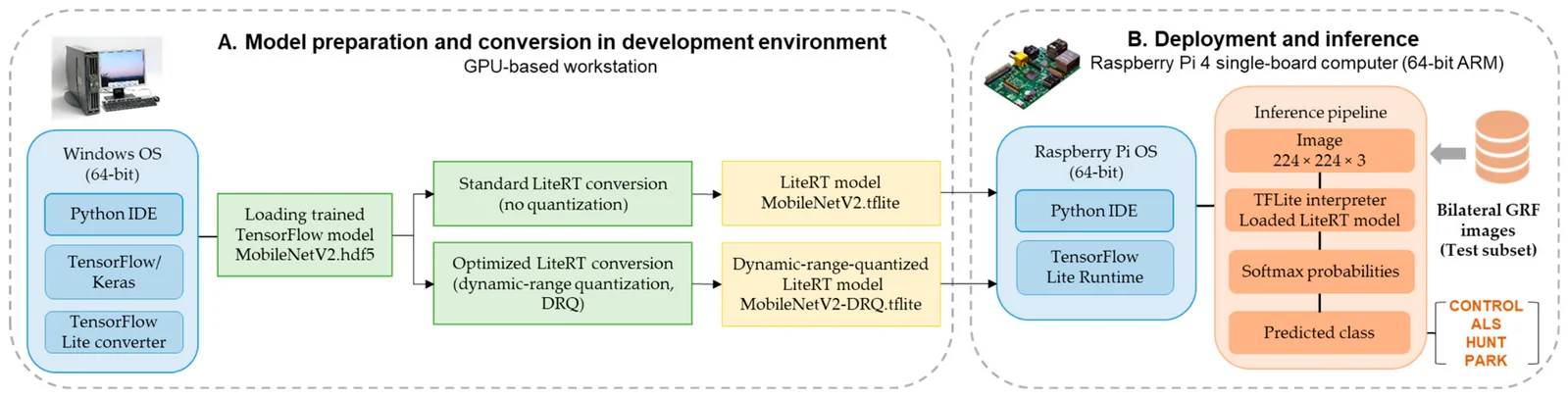
Workflow for converting and deploying the trained MobileNetV2 models on the Raspberry Pi 4. (**A**) Conversion of the TensorFlow/Keras models into standard and dynamic-range-quantized LiteRT formats in the development environment. (**B**) Deployment of the converted models on the Raspberry Pi 4 and inference using bilateral GRF images from the test subset. GRF: ground reaction force; DRQ: dynamic-range quantization; OS: operating system; IDE: integrated development environment.

To evaluate the feasibility of resource-constrained implementation, the trained MobileNetV2 models were deployed on a Raspberry Pi 4 single-board computer equipped with a quad-core ARM Cortex-A72 (ARMv8) 64-bit processor operating at 1.8 GHz and 8 GB of SDRAM. The device operated under the 64-bit Raspberry Pi OS (Debian Linux, version 12), and model inference was performed in a Python environment (version 3.11.2) using the TensorFlow Lite Runtime (version 2.21.0). The overall model conversion and deployment workflow is illustrated in Figure 3.

Both retrained and fine-tuned TensorFlow/Keras MobileNetV2 models were first loaded on the GPU-based workstation and converted to the LiteRT format, as illustrated in Figure 3A. Two versions of each model were generated. The first was obtained using standard LiteRT conversion without quantization, retaining the model parameters in 32-bit floating-point (float32) representation. The second was generated using the default LiteRT optimization setting (tf.lite.Optimize.DEFAULT), resulting in dynamic-range quantization (DRQ). In this configuration, model weights were quantized from float32 to 8-bit integer (int8) representation, thereby reducing model storage requirements, while model inputs and outputs remained in floating-point representation and activations were dynamically quantized during inference where applicable. The resulting standard and DRQ models were subsequently transferred to the Raspberry Pi 4 for on-device inference. Hereafter, these deployment configurations are referred to as TFLite and TFLite-DRQ, respectively.

For performance evaluation on the Raspberry Pi 4, the converted models were loaded using the TensorFlow Lite interpreter and evaluated using the bilateral GRF images from the test subset. Each 224 × 224 × 3 RGB image was provided to the corresponding MobileNetV2 model, which generated softmax probabilities for the CONTROL, ALS, HUNT, and PARK classes. The class associated with the highest probability was assigned as the predicted class. Classification performance of the TFLite and TFLite-DRQ models was compared with that obtained using the original TensorFlow/Keras models on the workstation to assess whether model conversion and DRQ affected predictive performance.

Resource efficiency on the Raspberry Pi 4 was evaluated in terms of model size, inference latency, incremental electrical power, and estimated incremental energy consumption per inference. Model size was determined from the storage size of the corresponding LiteRT model file. Inference latency t_Inf_ was measured separately for each image in the test subset and was defined as the time required by the Raspberry Pi 4 to perform a single forward pass of the MobileNetV2 model on a preloaded bilateral GRF image, including feature extraction and four-class classification. The bilateral GRF images were generated beforehand and provided as inputs to the network; therefore, the measured inference latency excluded bilateral GRF image generation, input loading, resizing, normalization, and input tensor preparation.

To estimate the additional electrical power associated with network inference, the board-level supply voltage and current were measured manually under idle conditions (U_Idle_, I_Idle_) and during inference (U_Inf_, I_Inf_) using a commercial USB voltage/current meter (Keweisi KWS-10VA), with resolutions of 0.01 V and 0.01 A, respectively. The incremental board-level power associated with inference was calculated as the difference between the power measured during inference and under idle conditions:

(10) $${∆P}_{\mathrm{Inf}}={P_{\mathrm{Inf}}-P_{\mathrm{Idle}}=U}_{\mathrm{Inf}}.I_{\mathrm{Inf}}-U_{\mathrm{Idle}}.I_{\mathrm{Idle}},$$

where P_Inf_ and P_Idle_ denote the board-level electrical power during inference and under idle conditions, respectively, and ΔP_Inf_ represents the incremental board-level power associated with inference. Subtracting the idle power reduces the contribution of baseline board consumption, allowing the additional power associated with network inference to be estimated.

The incremental energy consumption associated with a single inference was estimated from the calculated power difference and the corresponding inference latency:

E*_Inf_* = ∆P_Inf_.t*_Inf_*, (11)

where E_Inf_ denotes the estimated incremental energy consumption per inference.

## 3. Results

### 3.1. MobileNetV2 Classification Performance

According to the transfer-learning strategy illustrated in Figure 2, the models selected for test evaluation were those achieving the highest validation accuracy during the corresponding training stage. The epoch at which this maximum validation accuracy was reached varied substantially according to the MobileNetV2 configuration, transfer-learning stage, and GRF representation, ranging from 64 to 543 epochs. During classification-layer retraining, the higher-capacity model (α = 1.4) reached its highest validation accuracy considerably earlier (64–71 epochs) than the compact model (α = 0.35; 120–263 epochs), corresponding to approximately 2–4 times fewer epochs depending on the GRF representation. During fine-tuning, the highest validation accuracy was generally reached later, at 347–480 epochs for α = 0.35 and 202–543 epochs for α = 1.4. The effect of GRF representation was less consistent; however, the Occupancy Heatmap appeared to require longer optimization in some configurations, reaching its highest validation accuracy at epoch 263 for α = 0.35 during retraining and at epoch 543 for α = 1.4 during fine-tuning.

**Figure 4:**
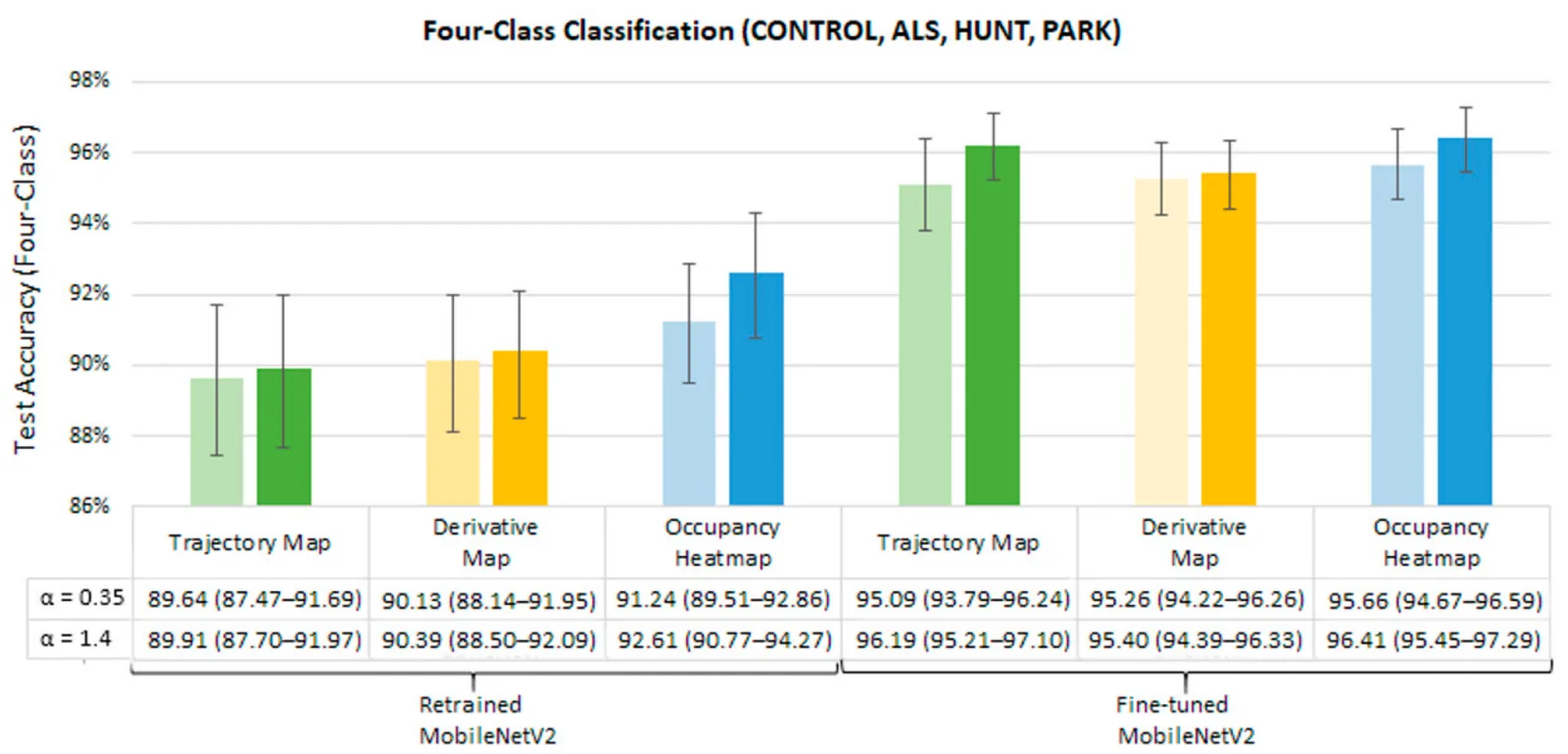
Overall four-class gait-cycle classification accuracy of the MobileNetV2 models on the test subset, using the Trajectory Map, Derivative Map, and Occupancy Heatmap representations. Results are shown separately for the retrained models (**left**) and fine-tuned models (**right**), with compact (α = 0.35, light bars) and higher-capacity (α = 1.4, dark bars) configurations. Error bars indicate 95% confidence intervals estimated using subject-clustered bootstrap resampling. The reported accuracies are based on gait-cycle-level data partitioning and do not represent subject-independent validation.

**Figure 5:**
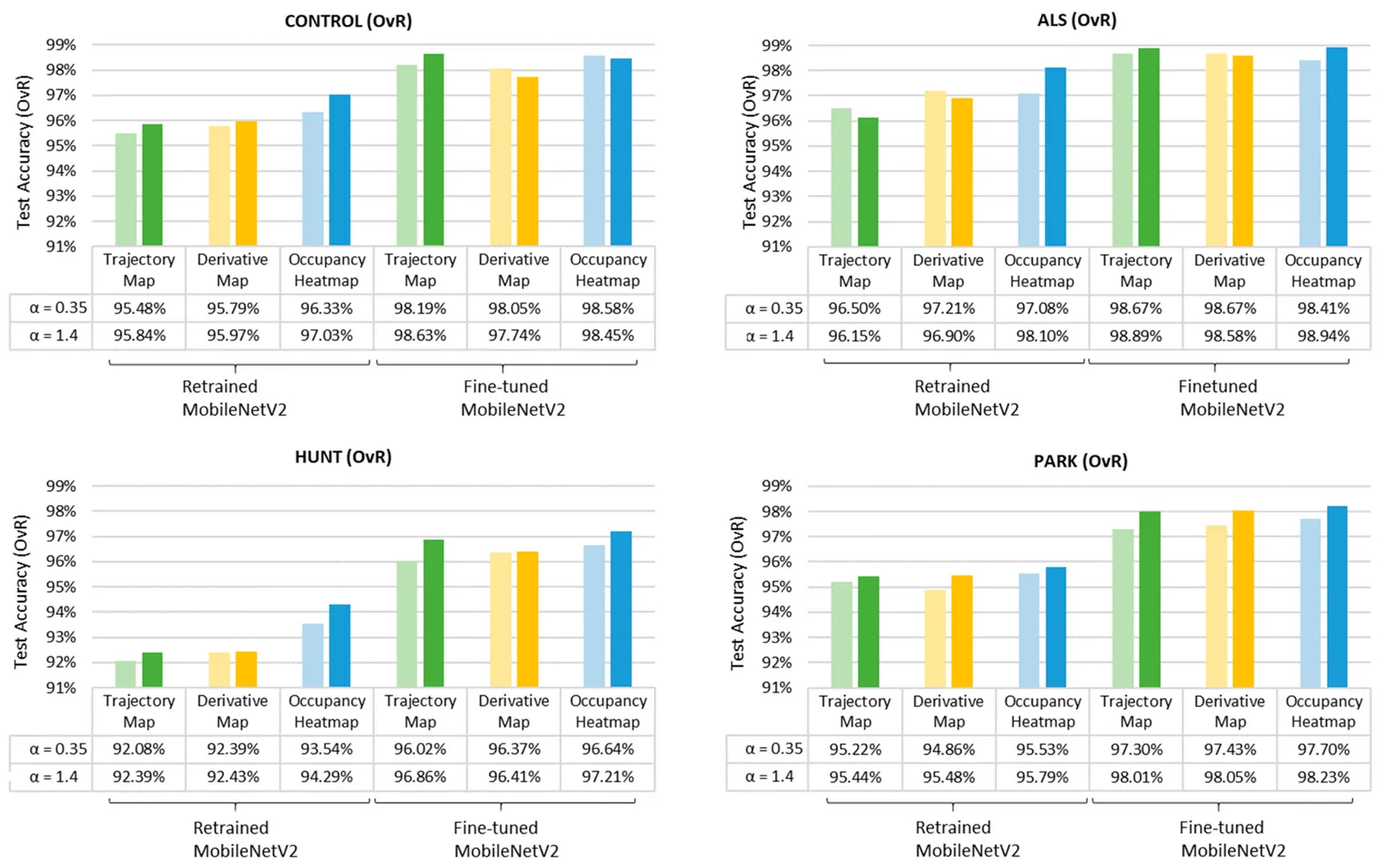
Class-specific one-vs-rest (OvR) classification accuracy of the MobileNetV2 models on the test subset for healthy controls (CONTROL), amyotrophic lateral sclerosis (ALS), Huntington’s disease (HUNT), and Parkinson’s disease (PARK). Results are presented for the three bilateral GRF image representations, separately for the retrained models (**left**) and fine-tuned models (**right**). Light and dark bars represent the compact (α = 0.35) and higher-capacity (α = 1.4) MobileNetV2 configurations, respectively. OvR accuracy is calculated separately for each diagnostic class and should not be interpreted as overall four-class classification accuracy.

The classification performance of the retrained and fine-tuned MobileNetV2 models on the test subset is summarized in Table 5 and Table 6, respectively. Class-specific precision, recall, specificity, F1-score, AUC, and OvR accuracy are reported for the CONTROL, ALS, HUNT, and PARK classes for each bilateral GRF representation and MobileNetV2 configuration. Overall four-class accuracy and the corresponding subject-clustered bootstrap 95% confidence intervals are reported in Figure 4, whereas the class-specific OvR accuracies are compared in Figure 5. The corresponding confusion matrices, together with macro-F1 and balanced accuracy, are provided in Section S2 of Supplementary Materials S1 (Tables S2 and S3). Subject-wise distributions of gait-cycle predictions and classification accuracy are reported in Section S4 of Supplementary Materials S1 (Tables S6–S10).

To further evaluate differences in overall four-class classification accuracy, paired comparisons were performed between MobileNetV2 configurations that differed in GRF image representation, network width multiplier, or training strategy. The comparisons included subject-clustered bootstrap 95% confidence intervals for accuracy differences and exploratory exact McNemar tests with Holm-adjusted *p*-values. Detailed results for all 24 comparisons are provided in Supplementary Table S1.

As shown in Figure 4, fine-tuning consistently improved overall four-class classification accuracy compared with retraining only the newly introduced classification layer. For the retrained models, accuracy ranged from 89.64% to 92.61%, corresponding to a maximum difference of approximately 3 percentage points across the three GRF representations and two MobileNetV2 configurations (α = 0.35 and α = 1.4). Following fine-tuning, accuracy increased to 95.09–96.41% across all representations and model configurations, and the maximum difference among configurations decreased to approximately 1.3 percentage points. Paired comparisons showed improvements following fine-tuning for all six matched configurations, with accuracy gains ranging from 3.81 to 6.29 percentage points and subject-clustered bootstrap 95% confidence intervals excluding zero (Supplementary Table S1). All six training-strategy comparisons also yielded Holm-adjusted *p*-values < 0.0001 in the exploratory exact McNemar tests. Among the retrained models with α = 1.4, the Occupancy Heatmap outperformed the Trajectory Map by 2.70 percentage points (95% CI: 0.99–4.51) and the Derivative Map by 2.21 percentage points (95% CI: 0.77–3.73), with Holm-adjusted *p*-values of 0.0024 and 0.0244, respectively. No network width comparison yielded a Holm-adjusted *p*-value below 0.05. Overall, the results provide consistent evidence of improved classification accuracy following fine-tuning, whereas differences between GRF representations and network width multipliers were less consistent across configurations.

The class-specific results in Table 5 and Table 6 and Figure 5 show that performance also varied among the diagnostic classes. For the retrained models, the lowest OvR accuracy was consistently obtained for HUNT (92.08–94.29%), compared with CONTROL (95.48–97.03%), ALS (96.15–98.10%), and PARK (94.86–95.79%). Across the six combinations of GRF representation and MobileNetV2 configuration, the difference between the highest and lowest class-specific OvR accuracies ranged from 3.54 to 4.82 percentage points. Following fine-tuning, OvR accuracy increased for all four classes: CONTROL (97.74–98.63%; +1.42 to +2.79 percentage points), ALS (98.41–98.94%; +0.84 to +2.74 percentage points), HUNT (96.02–97.21%; +2.92 to +4.47 percentage points), and PARK (97.30–98.23%; +2.08 to +2.57 percentage points). Although HUNT remained the class with the lowest OvR accuracy, it showed the largest improvement following fine-tuning, and the between-class spread decreased from 3.54–4.82 percentage points for the retrained models to 1.73–2.65 percentage points for the fine-tuned models. The corresponding AUC values were also consistently high after fine-tuning, ranging from 0.9900 to 0.9979.

Among the fine-tuned models (Table 6 and Figure 4), the Occupancy Heatmap with α = 1.4 achieved the highest observed overall four-class accuracy of 96.41% (95% CI: 95.45–97.29%). For this configuration, class-specific OvR accuracies were 98.45%, 98.94%, 97.21%, and 98.23% for CONTROL, ALS, HUNT, and PARK, respectively (between-class range: 1.73 percentage points). The corresponding F1-scores (97.14%, 96.71%, 95.71%, and 96.35%) and AUC values (0.9979, 0.9979, 0.9953, and 0.9971) indicated similarly high performance across the four classes. Although HUNT exhibited the lowest OvR accuracy, F1-score, and AUC for this configuration, the differences among the four classes remained relatively small.

Exploratory sex- and age-stratified analyses were additionally performed for the six fine-tuned MobileNetV2 configurations to assess potential demographic differences in class-specific recall. No statistically significant differences were identified after Holm adjustment within any model configuration (all adjusted *p*-values > 0.05). Detailed results and statistical methodology are provided in Section S3 of Supplementary Materials S1 (Tables S4 and S5).

### 3.2. Deployment Performance on Raspberry Pi 4

The deployment performance of the retrained and fine-tuned MobileNetV2 models on the Raspberry Pi 4 is summarized in Table 7 for the Trajectory Map, Derivative Map, and Occupancy Heatmap representations. Inference latency is reported as the mean ± standard deviation calculated across all gait-cycle images in the test subset. During the measurements, the Raspberry Pi 4 operated at a supply voltage of 5.24 V. The measured current was 0.39 A under idle conditions and 0.79 A during inference. These measurements corresponded to calculated board-level power values of approximately 2.04 W and 4.14 W, respectively. According to Equation (10), the incremental board-level power associated with inference was therefore estimated as 2.10 W. The estimated incremental energy per inference was subsequently calculated for each model configuration using this common power difference and the corresponding mean inference latency, according to Equation (11).

As seen in Table 7, model size and inference latency were strongly affected by the MobileNetV2 width multiplier. For the standard TFLite models, increasing α from 0.35 to 1.4 increased the model size from 1580 kB to 16,833 kB. On the Raspberry Pi 4, the compact α = 0.35 models required approximately 42–46 ms per inference, whereas the higher-capacity α = 1.4 models required approximately 324–374 ms, corresponding to approximately a 7.3–8.2-fold increase in inference latency. Consequently, the estimated incremental energy consumption ranged from approximately 89–97 mJ per inference for the compact α = 0.35 models, and from 680–785 mJ per inference for the higher-capacity α = 1.4 models. Thus, using the incremental board-level power associated with inference of 2.10 W, the higher-capacity configuration had an estimated incremental energy consumption approximately 7–8 times higher than the compact configuration.

Inference on the Raspberry Pi 4 was slower than on the GPU-based workstation, with the difference being considerably greater for the higher-capacity α = 1.4 models. The workstation values are therefore included primarily as a reference for the original TensorFlow/Keras implementation, whereas the Raspberry Pi 4 results characterize the computational requirements of the deployed models.

DRQ substantially reduced model storage requirements, decreasing the model size from 1580 to 595 kB for α = 0.35 and from 16,833 to 4780 kB for α = 1.4, corresponding to reductions of approximately 2.7 and 3.5 times, respectively. The effect of DRQ on inference latency was relatively small for the compact α = 0.35 models, whereas a more pronounced reduction was observed for the higher-capacity α = 1.4 models. Because all energy estimates were obtained using the same incremental board-level power of 2.10 W, differences in estimated incremental energy per inference directly reflected the corresponding differences in mean inference latency.

Model conversion to TFLite generally preserved the four-class classification accuracy obtained using the original TF/Keras models on the workstation, with only small differences observed between the two implementations (<1%). DRQ produced somewhat larger changes in classification performance, particularly for the retrained α = 1.4 models (up to 4%). In contrast, the fine-tuned models generally retained high classification accuracy after DRQ. Among the evaluated configurations, the fine-tuned α = 1.4 model using the Occupancy Heatmap achieved the highest four-class accuracy on the Raspberry Pi 4, reaching 96.41% with standard TFLite conversion and 96.19% after DRQ. The corresponding TFLite-DRQ model required 4780 kB of storage, with a mean inference latency of 343 ms and an estimated incremental energy consumption of approximately 720 mJ per inference.

### 3.3. Representative Examples of Correctly Classified and Misclassified Gait Cycles

**Figure 6:**
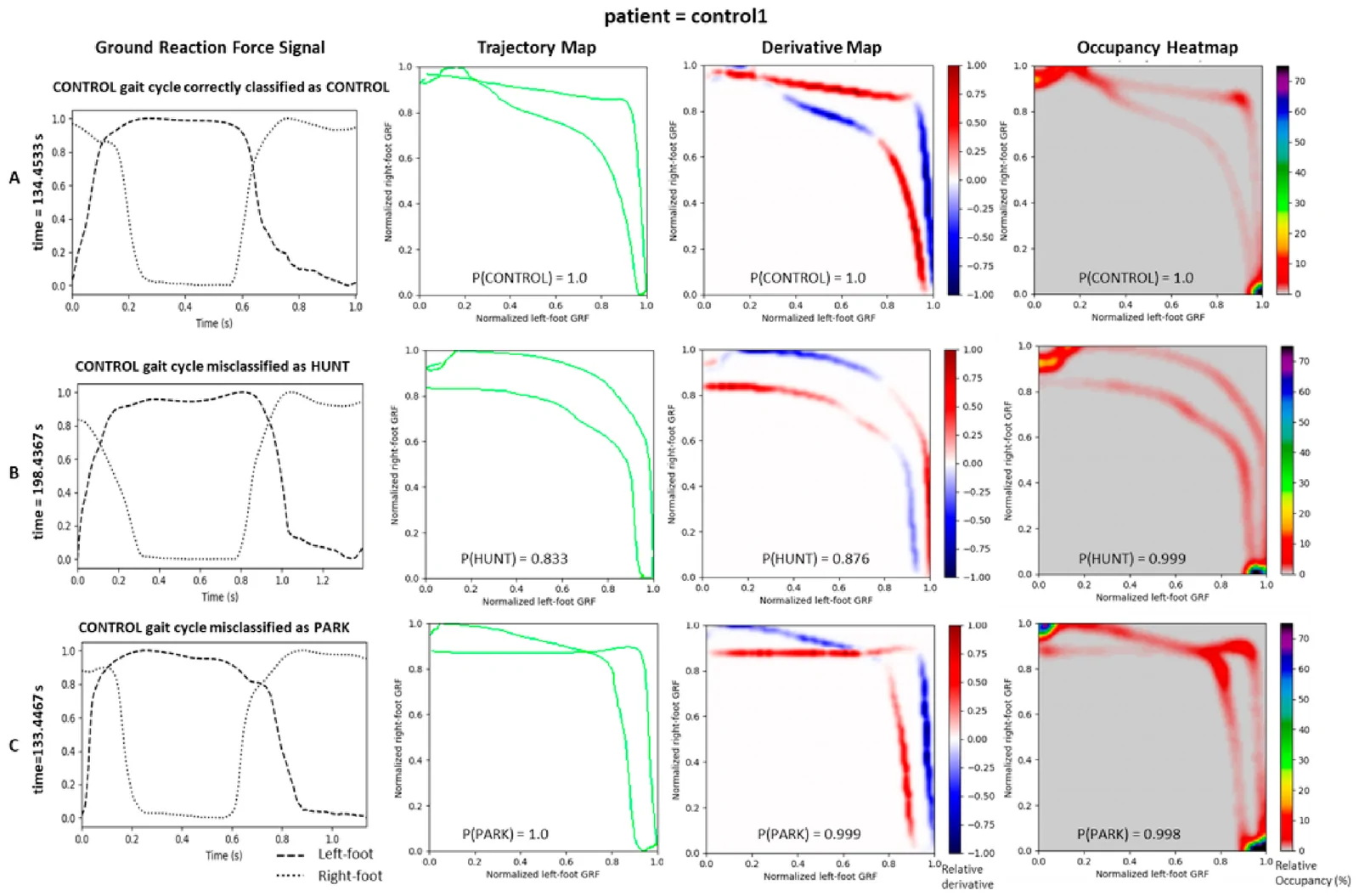
Representative correctly classified and misclassified gait cycles from subject control1 (CONTROL), evaluated using the fine-tuned MobileNetV2 model with α = 1.4. (**A**) Gait cycle correctly classified as CONTROL; (**B**) gait cycle misclassified as HUNT; (**C**) gait cycle misclassified as PARK. Each example shows the normalized bilateral GRF time series and the corresponding Trajectory Map, Derivative Map, and Occupancy Heatmap. The predicted diagnostic class and its associated maximum softmax probability, P(class), are indicated for each representation.

**Figure 7:**
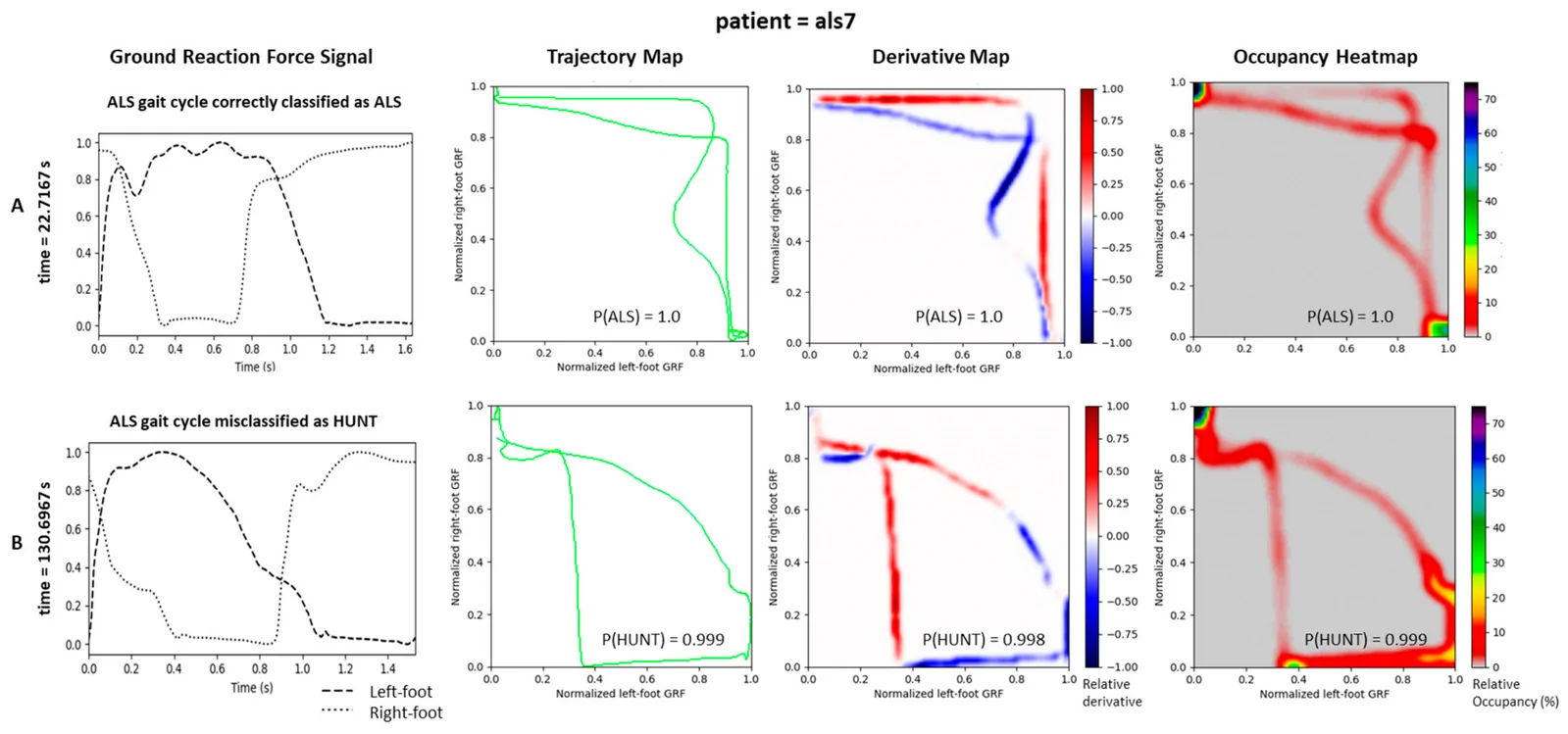
Representative correctly classified and misclassified gait cycles from subject als7 (ALS), evaluated using the fine-tuned MobileNetV2 model with α = 1.4. The examples illustrate variation in classification outcomes across gait cycles recorded from the same participant: (**A**) Gait cycle correctly classified as ALS; (**B**) gait cycle misclassified as HUNT. Normalized left- and right-foot GRF signals are shown alongside the corresponding Trajectory Map, Derivative Map, and Occupancy Heatmap. For each representation, the predicted diagnostic class and associated maximum softmax probability, P(class), are indicated.

**Figure 8:**
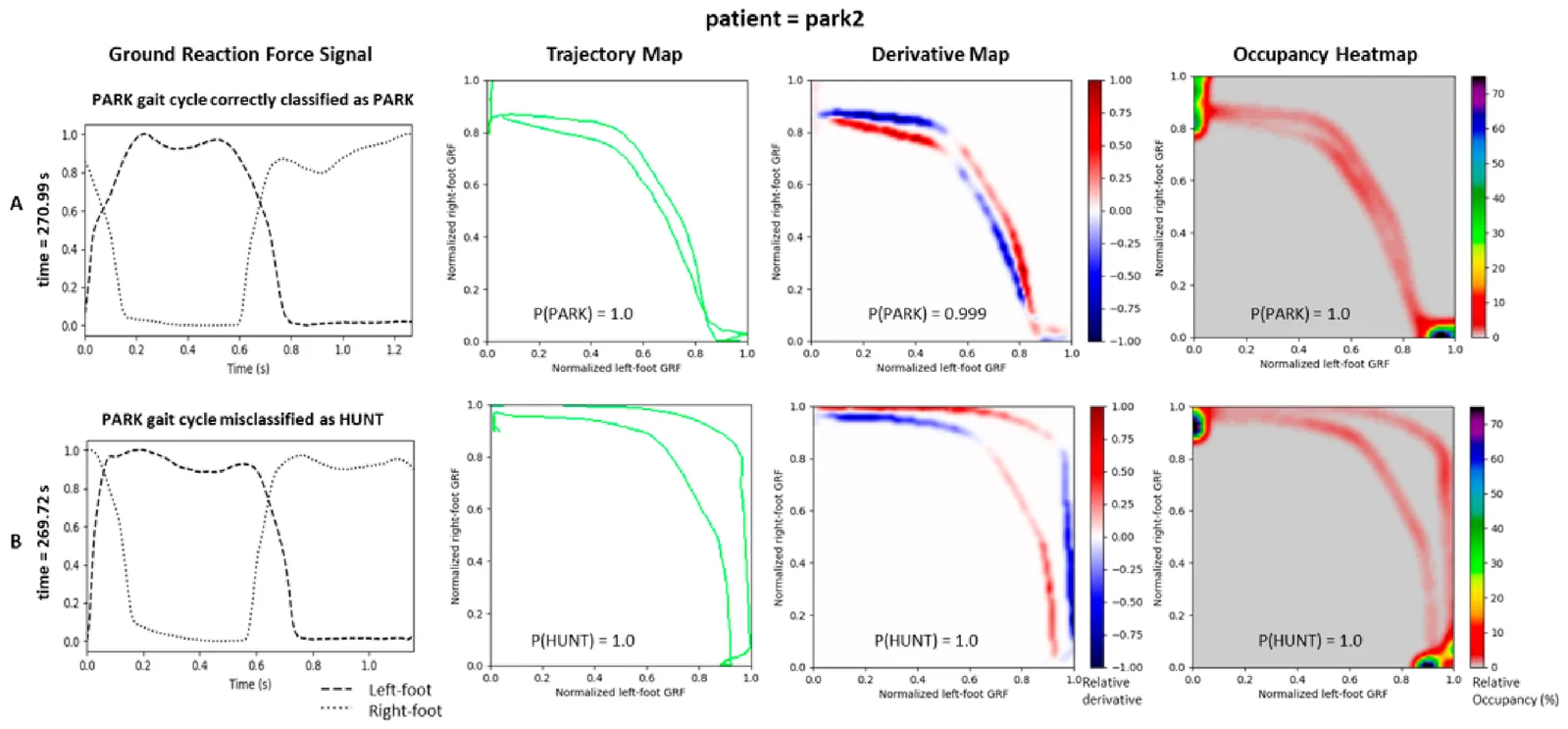
Representative correctly classified and misclassified gait cycles from subject park2 (PARK), evaluated using the fine-tuned MobileNetV2 model with α = 1.4. (**A**) Gait cycle correctly classified as PARK; (**B**) gait cycle misclassified as HUNT. Each example includes the normalized bilateral GRF time series and the corresponding Trajectory Map, Derivative Map, and Occupancy Heatmap. The predicted diagnostic class and associated maximum softmax probability, P(class), are indicated for each representation.

Representative examples of correctly classified and misclassified gait cycles are shown in Figure 6, Figure 7 and Figure 8 for the higher-capacity fine-tuned MobileNetV2 model (α = 1.4). The examples were selected to illustrate gait cycles for which the model produced consistent classification outcomes across the three input GRF representations. For each subject, a correctly classified gait cycle is presented together with one or more gait cycles that were consistently misclassified across the Trajectory Map, Derivative Map, and Occupancy Heatmap representations. Figure 6 shows three gait cycles from a CONTROL subject (patient ID: control1), including one correctly classified as CONTROL and two misclassified as HUNT and PARK, respectively. Figure 7 shows two gait cycles from an ALS subject (patient ID: als7), one correctly classified as ALS and one misclassified as HUNT. Figure 8 similarly shows two gait cycles from a PARK subject (patient ID: park2), one correctly classified as PARK and one misclassified as HUNT.

**Figure 9:**
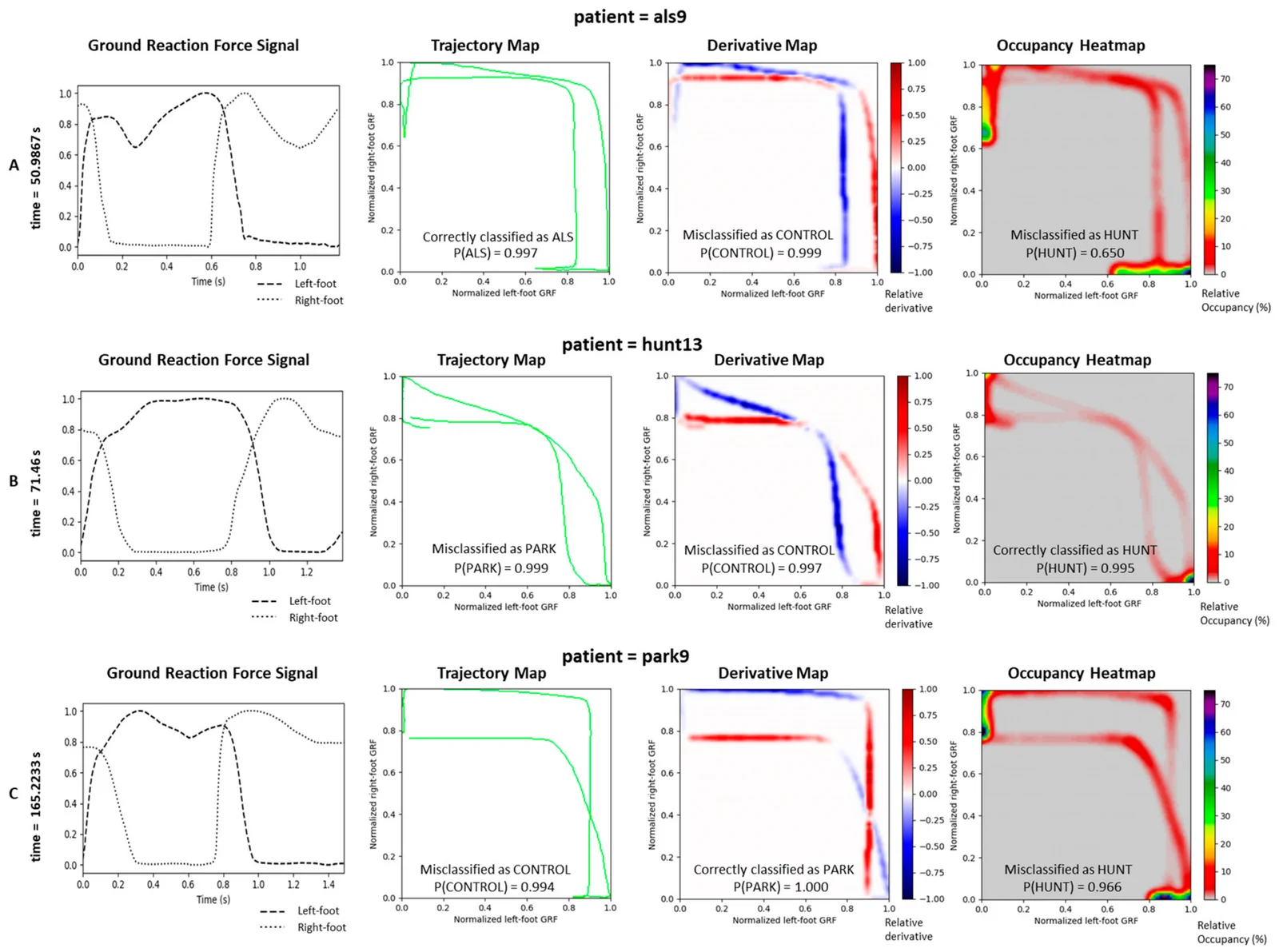
Representative gait cycles for which the three bilateral GRF image representations produced different classification outcomes using the fine-tuned MobileNetV2 model with α = 1.4. (**A**) ALS gait cycle correctly classified using the Trajectory Map but misclassified as CONTROL and HUNT using the Derivative Map and Occupancy Heatmap, respectively. (**B**) HUNT gait cycle correctly classified using the Occupancy Heatmap but misclassified as PARK and CONTROL using the Trajectory Map and Derivative Map, respectively. (**C**) PARK gait cycle correctly classified using the Derivative Map but misclassified as CONTROL and HUNT using the Trajectory Map and Occupancy Heatmap, respectively. Each example includes the normalized bilateral GRF time series and the three corresponding image representations. The predicted class and associated maximum softmax probability, P(class), are indicated for each representation.

These examples demonstrate that individual gait cycles from the same subject can produce different classification outcomes, while the three GRF representations may nevertheless yield consistent predictions for a given gait cycle. In contrast, Figure 9 presents three representative gait cycles for which the classification outcome differed among the three GRF representations. For the ALS example, only the Trajectory Map produced the correct classification; for the HUNT example, only the Occupancy Heatmap produced the correct classification; and for the PARK example, only the Derivative Map produced the correct classification. These examples illustrate that, for individual gait cycles, the classification outcome can depend on the GRF representation used. Possible factors contributing to these classification patterns are considered in Section 4.

## 4. Discussion

The present study introduced three complementary 2D representations of synchronized left- and right-foot GRF signals for multiclass gait classification. Rather than representing the two GRF signals independently, the proposed approach maps them into a common bilateral force space, thereby directly describing their synchronized relationship throughout the gait cycle. The Trajectory Map, Derivative Map, and Occupancy Heatmap emphasize complementary characteristics of this relationship, namely the evolution of the bilateral GRF trajectory, dynamic force transitions, and the distribution of bilateral loading states, respectively. An additional advantage of this formulation is that gait cycles of different durations are represented by images of identical dimensions without temporal resampling, while the complete gait cycle is represented without requiring alignment to a specific gait event. The classification results obtained with all three representations indicate that this bilateral force-space formulation retains information relevant to the discrimination among CONTROL, ALS, HUNT, and PARK.

In addition to evaluating classification performance, the study examined the feasibility of deploying the proposed approach on a resource-constrained computing platform, as computational efficiency represents an important consideration for its potential implementation in portable or edge-based gait-analysis systems. The Raspberry Pi 4 was used as an edge-computing platform to evaluate whether the trained MobileNetV2 models could perform on-device gait classification while reducing model storage requirements and maintaining practical inference latency and energy consumption. This deployment analysis complements the classification results by considering not only predictive performance but also the computational requirements associated with practical implementation. The classification performance and deployment characteristics observed in the present study are further discussed below in relation to the proposed GRF representations and MobileNetV2 configurations and compared with findings reported in previous studies.

### 4.1. MobileNetV2 Classification Performance

#### 4.1.1. Effects of Fine-Tuning, Model Capacity, and GRF Representation

The classification results demonstrate that all three bilateral GRF representations contain discriminative information relevant to differentiating CONTROL, ALS, HUNT, and PARK. Retraining only the newly introduced classification layer yielded overall four-class accuracies of 89.64–92.61% (Figure 4), indicating that the generic visual features learned by MobileNetV2 during ImageNet pretraining were transferable to the proposed GRF representations. Fine-tuning the pretrained feature extractor further increased overall four-class accuracy to 95.09–96.41% across all representations and model configurations. The corresponding subject-clustered bootstrap 95% confidence intervals for the individual models are presented in Figure 4. The paired comparisons showed improvements of 3.81–6.29 percentage points for all six matched configurations, with subject-clustered bootstrap 95% confidence intervals excluding zero and Holm-adjusted *p*-values below 0.0001 in the exploratory exact McNemar tests (Supplementary Table S1). These findings consistently support the benefit of adapting the pretrained feature extractor to the specific geometric, derivative, and occupancy patterns of the bilateral GRF images, rather than retraining only the classification layer.

The subject-wise analysis presented in Supplementary Materials S1 (Tables S6–S10) provides additional insight into the distribution of gait-cycle classification performance across individual subjects. The mean subject-wise accuracy ranged from 89.34% to 92.32% for the retrained models and from 94.88% to 96.25% for the fine-tuned models, closely corresponding to the overall gait-cycle accuracies of 89.64–92.61% and 95.09–96.41%, respectively (Figure 4). This agreement indicates that the overall improvement associated with fine-tuning was also reflected in the equally weighted mean accuracy across subjects. Nevertheless, the subject-wise results revealed variability in classification performance among individual participants, with minimum accuracies ranging from 47.06% to 66.67% for the retrained models and from 76.19% to 83.33% for the fine-tuned models. These findings should not be interpreted as evidence of generalization to previously unseen subjects, because the dataset was partitioned at the gait-cycle level.

The influence of MobileNetV2 model capacity was less pronounced than that of the training strategy. The compact α = 0.35 and higher-capacity α = 1.4 configurations contained 415,332 and 4,370,884 parameters, respectively (Table 3), representing an approximately 10.5-fold difference in total parameter count. Nevertheless, the fine-tuned models achieved overall four-class accuracies within the relatively narrow range of 95.09–96.41% (Figure 4). Increasing the width multiplier produced observed accuracy differences ranging from 0.13 to 1.37 percentage points across the six matched comparisons (Supplementary Table S1). Although the bootstrap 95% confidence interval excluded zero for the fine-tuned Trajectory Map comparison (+1.11 percentage points; 95% CI: 0.30–1.96), its Holm-adjusted McNemar *p*-value was 0.0687. None of the six width comparisons yielded a Holm-adjusted *p*-value below 0.05. Thus, the present results do not establish a consistent classification advantage for the higher-capacity configuration, despite its substantially greater number of parameters. However, the absence of statistically significant differences should not be interpreted as evidence of equivalence or non-inferiority of the compact models.

The three bilateral GRF representations also produced relatively similar classification performance after fine-tuning, although the Occupancy Heatmap achieved the highest observed overall four-class accuracy of 96.41% (95% CI: 95.45–97.29%) with α = 1.4 (Figure 4). Among the retrained models with α = 1.4, the Occupancy Heatmap exceeded the Trajectory Map by 2.70 percentage points (95% CI: 0.99–4.51; Holm-adjusted *p* = 0.0024) and the Derivative Map by 2.21 percentage points (95% CI: 0.77–3.73; Holm-adjusted *p* = 0.0244). However, none of the representation comparisons among fine-tuned models yielded a Holm-adjusted *p*-value below 0.05 (Supplementary Table S1). For example, the fine-tuned Occupancy Heatmap with α = 1.4 exceeded the Derivative Map by 1.02 percentage points (95% CI: 0.14–1.89), but the corresponding Holm-adjusted *p*-value was 0.2499. These findings indicate that the observed advantage of the Occupancy Heatmap depends on the training strategy and model configuration and do not establish its general superiority. Nevertheless, its representation of the frequency with which different bilateral loading states occur may provide useful discriminative information beyond trajectory geometry alone. Taken together, the results suggest that trajectory geometry, local force transitions, and bilateral-state occupancy provide partly overlapping but potentially complementary descriptions of gait.

#### 4.1.2. Class-Specific Classification Performance

The class-specific results provide additional insight into the multiclass classification task. HUNT was consistently the most difficult class to discriminate in the retrained models, with OvR accuracies of 92.08–94.29%, compared with 95.48–97.03% for CONTROL, 96.15–98.10% for ALS, and 94.86–95.79% for PARK (Table 5 and Figure 5). Fine-tuning improved OvR accuracy for all four classes, with the largest improvement observed for HUNT (+2.92 to +4.47 percentage points), and reduced the between-class spread from 3.54–4.82 percentage points in the retrained models to 1.73–2.65 percentage points in the fine-tuned models (Table 6 and Figure 5). For the best-performing fine-tuned Occupancy Heatmap model with α = 1.4, the OvR accuracies were 98.45%, 98.94%, 97.21%, and 98.23% for CONTROL, ALS, HUNT, and PARK, respectively, while the corresponding F1-scores were 97.14%, 96.71%, 95.71%, and 96.35%, and the AUC values ranged from 0.9953 to 0.9979 (Table 6). Thus, the high overall four-class accuracy was supported by consistently high class-specific performance rather than being driven by a single diagnostic group.

Nevertheless, HUNT remained the most difficult class to discriminate after fine-tuning, although its OvR accuracy remained high (96.02–97.21%). This may reflect the substantial cycle-to-cycle variability of Huntington’s gait, as choreic movements are intermittent and may affect individual gait cycles to different degrees. Moreover, gait abnormalities such as altered stride dynamics and increased gait variability can occur across NDDs, potentially resulting in overlapping GRF characteristics between individual HUNT gait cycles and gait cycles from the other classes. Because classification in the present study was performed at the individual gait-cycle level, such variability and overlap may limit the discriminative information available within a single cycle. This interpretation is also consistent with the representative misclassified gait cycles discussed in Section 4.2. Nevertheless, HUNT showed the largest improvement following fine-tuning (+2.92 to +4.47 percentage points), contributing to the reduced performance differences among the four classes.

#### 4.1.3. Exploratory Sex- and Age-Stratified Performance

Exploratory sex- and age-stratified analyses of the six fine-tuned MobileNetV2 configurations yielded observed differences in class-specific recall ranging from −3.17 to +3.19 percentage points between male and female participants and from −5.33 to +3.51 percentage points between younger and older participants (Supplementary Materials S1, Tables S4 and S5). The age subgroups were defined using the diagnosis-specific median age as the cutoff (CONTROL: 35 years; ALS: 62 years; HUNT: 44.5 years; PARK: 68 years), with participants aged at or below the median classified as younger and those above the median as older. None of the subgroup comparisons were statistically significant after Holm adjustment for multiple comparisons (sex: adjusted *p* = 0.678–1.000; age: adjusted *p* = 0.202–1.000). However, these findings should be interpreted cautiously because of the limited subgroup sizes and the uneven sex distribution across diagnostic categories, particularly within CONTROL (2 male and 14 female participants) and ALS (10 male and 3 female participants). These characteristics may limit the statistical power to detect subgroup differences, and the absence of statistically significant differences does not establish equivalent classification performance across demographic groups. Further evaluation using larger and more demographically balanced cohorts is therefore warranted.

### 4.2. Representative Examples of Correctly Classified and Misclassified Gait Cycles

The representative examples in Figure 6, Figure 7 and Figure 8 support this interpretation at the individual gait-cycle level. Gait cycles originating from the same subject could be either correctly classified or consistently misclassified across all three GRF representations. For example, individual CONTROL cycles were classified as HUNT or PARK, whereas examples from ALS and PARK were misclassified as HUNT. Because the diagnostic label applies to the subject, whereas classification in the present study was performed independently for each gait cycle, individual cycles may not express disease-related gait characteristics to the same degree. The observed errors may therefore reflect within-subject gait variability in addition to limitations of the classifier. This distinction is relevant for future practical applications, in which classification based on multiple gait cycles from the same subject could potentially provide more robust diagnostic information than decisions based on individual gait cycles.

Figure 9 provides a complementary perspective by showing examples in which the three GRF representations produced different predictions for the same gait cycle. In the selected ALS, HUNT, and PARK examples, the correct prediction was obtained from the Trajectory Map, Occupancy Heatmap, and Derivative Map, respectively, while the remaining representations produced erroneous classifications. These examples reinforce the interpretation that the three transformations are not merely alternative visualizations of identical information. Because each representation emphasizes a different property of the synchronized GRF signals, classification-relevant characteristics may be more apparent in one representation than another for a particular gait cycle. This observation suggests a potentially useful direction for future work: rather than selecting a single GRF representation, predictions or learned features from multiple representations could be combined through ensemble or multimodal fusion strategies. Whether such fusion improves classification performance and subject-level generalization would, however, require independent evaluation.

### 4.3. Deployment Performance on Raspberry Pi 4

From an implementation perspective, the Raspberry Pi 4 experiments demonstrate that the proposed approach can be executed on a resource-constrained single-board computer. Standard TFLite conversion generally preserved the classification performance of the original TensorFlow/Keras models, indicating that model conversion and on-device deployment introduced only small changes in predictive performance. Dynamic-range quantization substantially reduced model storage requirements by approximately 62% for α = 0.35 and 72% for α = 1.4, while the fine-tuned models generally retained high classification accuracy (Table 7). These findings are relevant for edge-based applications in which model storage, inference latency, and estimated incremental energy consumption represent important implementation constraints.

A clear trade-off was observed between MobileNetV2 capacity and resource requirements. The compact α = 0.35 models required approximately 42–46 ms per inference on the Raspberry Pi 4, compared with approximately 324–374 ms for the higher-capacity α = 1.4 models, while the corresponding estimated incremental energy consumption ranged from approximately 89–97 mJ and 680–785 mJ per inference, respectively (Table 7). Despite the approximately tenfold difference in model parameter count and the substantial differences in computational requirements, the observed differences in classification accuracy after fine-tuning remained comparatively small. Where computational efficiency is prioritized, the compact fine-tuned models therefore provide a favorable performance–resource trade-off, achieving high observed accuracy with substantially lower inference latency and estimated energy consumption. Although the higher-capacity models achieved modestly higher observed accuracies in the matched configurations, none of the six width comparisons yielded a Holm-adjusted *p*-value below 0.05 (Supplementary Table S1). These findings support considering the compact configurations for resource-constrained deployment, without establishing their statistical equivalence or non-inferiority in classification performance. The reported energy values represent estimates of incremental board-level energy associated with inference, based on a common measured power difference, rather than direct energy measurements for individual model configurations.

Among the evaluated configurations, the fine-tuned α = 1.4 Occupancy Heatmap model illustrates the highest-performance end of this trade-off, achieving 96.41% four-class accuracy with standard TFLite and 96.19% after dynamic-range quantization, with the latter requiring approximately 4.78 MB of storage, 343 ms per inference, and an estimated incremental energy consumption of 720 mJ per inference (Table 7). In contrast, all compact α = 0.35 fine-tuned TFLite and TFLite-DRQ models maintained four-class accuracies above 94% while substantially reducing model size, inference latency, and estimated energy consumption. Thus, rather than identifying a single deployment configuration as universally preferable, the results characterize different operating points within the performance–resource trade-off, allowing the model configuration to be selected according to the constraints of the intended application.

### 4.4. Comparison with Published Studies

#### 4.4.1. Classification Performance and Methodological Comparison

The classification performance obtained in the present study was compared with previously published multiclass approaches evaluated using the Gait in Neurodegenerative Disease Database (Table 8). The reported results should be interpreted with caution because the studies differ considerably in signal representation, input duration, feature extraction, classifier architecture, calculation of the accuracy metrics and validation strategy. Consequently, differences in reported classification performance cannot be attributed solely to the classification method.

The studies summarized in Table 8 differ in their validation protocols. These differences are particularly important when interpreting results obtained from the relatively small number of individuals represented in the GNDD database [58]. We identified explicit evidence of patient-independent evaluation in two studies [36,39], in which test subjects were excluded from training. For the remaining studies [22,25,31,35,38,40,41,42,44,56], subject-wise separation between training and test data could not be established unambiguously from the reported validation procedures. Although several of these studies employed cross-validation, this does not necessarily ensure patient-independent evaluation unless partitioning is performed at the subject level. In the present study, random gait-cycle-level partitioning was used. Consequently, the reported test accuracy reflects classification of previously unseen gait cycles rather than generalization to previously unseen subjects and should not be directly compared with results obtained using subject-independent validation.

Zeng et al. (2026) [39] illustrate the influence of validation protocols on reported performance. Their study reported accuracies of approximately 99.6% under random sample-level partitioning, 99.5% under fivefold cross-validation, 98.3% under leave-one-gait-cycle-out evaluation, and 98.1% under patient-level holdout testing. The reduction observed under patient-level holdout testing (approximately 0.1–1.5 percentage points) illustrates the importance of distinguishing patient-dependent from patient-independent evaluation. However, these findings are specific to the experimental settings of Zeng et al. and do not establish the corresponding effect of subject-independent validation on the present approach.

An additional source of heterogeneity is the definition of classification accuracy. Some studies report overall four-class accuracy [22,25,35,36,39], whereas others present class-specific one-vs-rest accuracy [25,36,40,41,44] or do not define the reported metric sufficiently clearly to permit an unambiguous classification [31,38,42]. One-vs-rest accuracy includes true negatives from the remaining diagnostic groups and may therefore be substantially higher than the corresponding overall four-class accuracy. In the present study, the overall four-class accuracies of the fine-tuned MobileNetV2 models ranged from 95.1% to 96.4%, whereas their class-specific one-vs-rest accuracies ranged from 96.0% to 98.9%. These metrics represent different performance measures, and one-vs-rest accuracy should not be used as a substitute for overall multiclass accuracy. Table 8 distinguishes these accuracy definitions, where they can be established from the original publications. Differences in metric definitions and validation protocols must therefore be considered when interpreting the reported results.

Several studies have investigated multiclass neurodegenerative gait classification using handcrafted temporal, statistical, or nonlinear gait features combined with conventional machine-learning classifiers. Among approaches based on features extracted from individual gait cycles, Erdaş et al. (2023) [22] reported overall four-class accuracies below 70% using gait timing features available in the GNDD database [58], whereas Mengarelli et al. (2022) [25] reported an overall four-class accuracy of 94.84% using time-domain and time-dependent spectral descriptors. Other studies extracted features from longer signal segments. Ozeloglu et al. (2024) [35] reported an overall four-class accuracy of 93.5% using detrended fluctuation analysis, dynamic time warping, and autocorrelation features derived from 10-s GRF segments. Nam Nguyen et al. (2020) [31] evaluated statistical and multiscale sample entropy features extracted from GRF segments lasting 10–60 s, reporting an accuracy of 99.68% using a 10-s window and KNN. These studies demonstrate the range of approaches and reported performance values obtained using handcrafted features, although their experimental settings differ.

Other studies have employed neural networks trained from scratch using GRF signals or image representations derived from gait features. Modaresnia et al. (2024) [38] reported overall four-class accuracies of 70.4–75.1% using BiLSTM networks applied to 30-s GRF segments from the left foot, right foot, or both feet. In their experimental setting, combining the left- and right-foot signals yielded higher accuracy than using the left-foot signal alone but did not outperform the right-foot input. Erdaş et al. (2024) [40] reported a one-vs-rest accuracy of 84.7% using a CNN applied to QR-code representations of swing, stance, and double-support intervals. More recently, Zeng et al. (2026) [39] proposed a dual-branch network incorporating convolutional and attention mechanisms for gait-cycle-level classification and reported high performance under several validation protocols, including patient-level holdout testing that excluded test subjects from training. Although the present study obtained higher reported accuracies than some of these approaches, differences in experimental settings preclude attributing those differences to the proposed representation or transfer-learning strategy alone.

A number of studies have transformed GRF signals into two-dimensional representations suitable for analysis using pretrained convolutional neural networks, reporting accuracy values ranging from 92.7% to 99.9% under different evaluation protocols and accuracy definitions [38,41,42,44]. Lin et al. (2020) [44] used recurrence plots generated from 10-s GRF segments, whereas Setiawan et al. (2021) [41] employed time-frequency spectrograms derived from GRF segments of different durations (10–60 s), with AlexNet used for feature extraction. Modaresnia et al. (2024) [38] combined spectrogram representations of 30-s GRF segments with pretrained AlexNet, GoogLeNet, and VGG16 architectures, reporting overall four-class accuracies approaching 100%. Of particular relevance to the present study, Torghabeh et al. (2024) [42] incorporated bilateral gait information through wavelet-coherence representations of left- and right-foot GRF signals. Their approach used 5- or 10-s signal windows containing multiple gait cycles and pretrained CNN architectures, including AlexNet, GoogLeNet, and SqueezeNet, with reported accuracies ranging from 92.7% to 99.2%. In contrast, the present approach maps synchronized bilateral GRF signals directly into a common two-dimensional force space and performs classification using individual gait cycles. This representation provides a different means of encoding bilateral loading relationships without requiring time-frequency transformations, recurrence analysis, or manually engineered gait descriptors. Nevertheless, its relative advantages over previously published encoding approaches remain to be established through controlled comparisons under identical experimental conditions.

Overall, Table 8 illustrates the diversity of handcrafted and image-based approaches used for multiclass neurodegenerative gait classification. Within this context, the present method achieved high overall four-class classification accuracy using synchronized bilateral GRF signals from individual gait cycles. Its principal methodological distinction is the combination of single-gait-cycle analysis, bilateral force-space representations, and transfer learning with MobileNetV2, rather than a demonstrated performance advantage over previously published methods.

#### 4.4.2. Comparison with Embedded Gait-Analysis Systems

From the perspective of edge implementation, the deployment results of the present study can also be considered in relation to previously reported embedded gait-analysis systems (Table 9). Direct numerical comparison should, however, be made cautiously because the reported studies address different classification tasks, use different sensing modalities and input durations, and employ hardware platforms with substantially different computational capabilities. Most previous embedded implementations focused specifically on FoG detection in Parkinson’s disease using wearable inertial or multimodal sensors, whereas the present study addresses four-class NDD classification from bilateral GRF representations. Reported processing times therefore reflect not only differences in classifier complexity but also differences in preprocessing, input duration, hardware, and the definition of inference latency. Notably, Saadeh et al., 2017 [56], the only identified study reporting an FPGA implementation for a comparable four-class NDD classification task (Table 8), did not report model size or inference time and therefore could not be included in the deployment comparison presented in Table 9.

Table 9 illustrates the broad range of computational requirements achievable for edge-based gait analysis. Gokul et al. (2020) [48] implemented a highly compact ProtoNN classifier on an ATMega2560, requiring approximately 20 ms for classifier execution; when feature extraction was included, the minimum total ML-subsystem latency increased to 44.5 ms. Hou et al. (2023) [49] deployed an 8-bit quantized depth-wise CNN for multimodal FoG detection on a low-power microcontroller, with an inference time below 2.3 s for a 3-s input window, whereas Slemenšek et al. (2024) [50] reported an average FoG detection delay of 261 ms for a CNN + RNN-based system implemented on an Arduino Portenta H7. Al-Adhaileh et al. (2025) [51] similarly demonstrated edge deployment of a quantized deep-learning FoG detector, reporting inference latency below 350 ms on platforms including the Raspberry Pi.

Against this heterogeneous background, the present MobileNetV2 implementations achieved inference times of approximately 42–46 ms for α = 0.35 and 324–374 ms for α = 1.4 on the Raspberry Pi 4 (Table 9). The compact models therefore operated within the lower range of the reported edge-processing times while retaining high four-class classification performance. The higher-capacity models required substantially greater computational time but remained within a sub-second inference range. Together with the reduction in model size obtained through dynamic-range quantization, these results demonstrate that the proposed bilateral GRF representations and MobileNetV2 classifiers can be deployed across different performance–resource operating points. However, because the systems summarized in Table 9 differ substantially in task, sensing modality, input window, hardware, and latency definition, the comparison should be interpreted as evidence of the practical scale of edge implementation rather than as a direct performance benchmark.

### 4.5. Limitations of the Study

Several limitations of the present study should be considered. First, the analysis was performed using a relatively small publicly available database comprising 64 subjects, despite the much larger number of extracted gait cycles. Furthermore, differences in demographic characteristics among the diagnostic groups, including age and sex distribution, may represent potential confounding factors that cannot be separated from disease-related gait characteristics in the present dataset. Moreover, the small and unevenly distributed sex- and age-specific subgroups limit the statistical power of the exploratory demographic analyses, particularly for diagnostic categories with pronounced sex imbalance. Consequently, the absence of statistically significant subgroup differences after correction for multiple comparisons does not establish equivalent classification performance across demographic groups. In addition, the adopted random gait-cycle-level partitioning allowed gait cycles from the same subject to occur in the training, validation, and test subsets. Consequently, the reported test performance primarily evaluates classification of previously unseen gait cycles rather than generalization to completely unseen subjects. Since gait cycles obtained from the same individual may share subject-specific characteristics, neither the overall gait-cycle accuracy nor the subject-wise accuracy summaries should be interpreted as an estimate of diagnostic performance in independent patients. Future studies should therefore evaluate the proposed representations using subject-independent partitioning or leave-one-subject-out validation and, preferably, external datasets. Additionally, the experiments were based on a single dataset partition and one training run per model configuration. Although subject-clustered bootstrap confidence intervals were used to quantify uncertainty in the reported test performance, they do not capture variability arising from different dataset partitions or random training initializations. Furthermore, the paired bootstrap confidence intervals were not adjusted for multiple comparisons, whereas the exploratory exact McNemar tests used Holm adjustment separately within the three comparison families. Because these tests treated gait-cycle predictions as independent observations, their *p*-values did not account for within-subject dependence and should therefore be interpreted cautiously. Accordingly, the statistical comparisons should be considered exploratory, and the absence of statistically significant differences between model configurations should not be interpreted as evidence of equivalence or non-inferiority, which would require dedicated analyses with prespecified performance margins.

A second limitation is that classification was performed at the individual gait-cycle level. Although this design enabled detailed analysis of within-subject classification variability, a practical gait-assessment system would normally have access to multiple consecutive gait cycles. Aggregating predictions over several cycles could potentially reduce the influence of atypical individual cycles and provide a more stable subject-level decision. In addition, the current study considered only vertical bilateral GRF signals and a single dataset acquired under controlled walking conditions. Evaluation using additional datasets, acquisition systems, disease severities, and real-world walking conditions will be necessary to determine the robustness and generalizability of the proposed representations.

Although the three proposed image representations were evaluated under the same experimental settings, no direct experimental comparison with previously published GRF-to-image encoding methods was performed. In addition, controlled comparisons with a 1D CNN operating on raw bilateral GRF signals, single-foot GRF inputs, and MobileNetV2 trained from scratch were not conducted, limiting conclusions about the relative benefits of the proposed image representations, bilateral signal information, and ImageNet pretraining. Further investigation is also needed to determine whether alternative strategies for combining the complementary information captured by these representations could improve classification performance.

## 5. Conclusions

This study introduced three complementary 2D representations of synchronized left- and right-foot GRF signals—the Trajectory Map, Derivative Map, and Occupancy Heatmap—for four-class discrimination among CONTROL, ALS, HUNT, and PARK gait cycles. By mapping the synchronized GRF signals into a common bilateral force space, the proposed approach represents different aspects of bilateral gait dynamics while producing fixed-size image inputs independently of gait-cycle duration and without requiring temporal resampling. Transfer learning with MobileNetV2 demonstrated that all three representations contained discriminative information, with fine-tuning increasing the overall four-class accuracy to 95.09–96.41%. Paired comparisons consistently supported the benefit of fine-tuning, with improvements of 3.81–6.29 percentage points across all six matched configurations (Holm-adjusted *p* < 0.0001 in all six exploratory exact McNemar tests). The Occupancy Heatmap achieved the highest observed overall four-class accuracy (96.41%; 95% CI: 95.45–97.29%), although its advantage over the other representations was not consistently supported across model configurations after adjustment for multiple comparisons. Exploratory sex- and age-stratified analyses did not identify statistically significant differences in class-specific recall after adjustment for multiple comparisons; however, the limited and uneven subgroup sizes do not allow potential demographic confounding to be excluded. The relatively similar performance obtained across the three representations and the representation-dependent classification outcomes observed for individual gait cycles suggest that trajectory geometry, dynamic force transitions, and bilateral-state occupancy may provide partly overlapping and potentially complementary information. These findings motivate future investigation of fusion strategies combining multiple representations and aggregation of predictions across multiple gait cycles.

Deployment on the Raspberry Pi 4 further demonstrated the feasibility of performing the proposed classification on a resource-constrained computing platform. The compact MobileNetV2 configuration (α = 0.35) maintained high classification performance while requiring substantially lower inference latency and estimated energy consumption than the higher-capacity α = 1.4 configuration, whereas dynamic-range quantization considerably reduced model storage requirements with only small changes in classification performance. Although the higher-capacity configurations achieved modestly higher observed accuracies, none of the six paired comparisons between the two MobileNetV2 width multipliers yielded a Holm-adjusted *p*-value below 0.05 in the exploratory exact McNemar tests. Together, these findings support the practical relevance of compact models for resource-constrained implementation, although equivalence or non-inferiority in classification performance was not established. The proposed framework therefore provides different operating points for balancing predictive performance and computational requirements in future portable or edge-based gait-analysis systems. Nevertheless, because the present evaluation used gait-cycle-level data partitioning, the reported performance should be interpreted as classification of previously unseen gait cycles rather than independent patients. Subject-independent validation, preferably using external datasets and multiple-cycle subject-level decision strategies, is therefore an essential next step toward assessing the generalizability and practical diagnostic potential of the proposed approach.

## Figures and Tables

**Table 1:** Demographic characteristics of the subjects included in the Gait in Neurodegenerative Disease Database (Version 1.0.0) [58], divided into four diagnostic classes—CONTROL: Healthy controls, ALS: amyotrophic lateral sclerosis, HUNT: Huntington’s disease, PARK: Parkinson’s disease. The last column reports the number of gait cycles per recording for which temporal gait features are available in the corresponding time-series file (.ts). Continuous variables are presented as the mean value ± standard deviation.

Diagnosis	Nb Subjects	Age (Years)	Height (m)	Weight (kg)	Gender	Gait Cycles
CONTROL	16	39 ± 19	1.83 ± 0.08	67 ± 11	12.5% male	255 ± 18
ALS	13	56 ± 13	1.74 ± 0.10	77 ± 21	76.9% male	196 ± 38
HUNT	20	47 ± 13	1.83 ± 0.11	72 ± 17	30% male	242 ± 30
PARK	15	67 ± 11	1.87 ± 0.15	75 ± 17	66.7% male	246 ± 24

**Table 2:** Summary of the bilateral GRF representations investigated in this study.

Representation	Short Name	Representation Principle	Main Gait Characteristic
Bilateral GRF Trajectory Map	Trajectory Map	Continuous trajectory formed by synchronized bilateral GRF samples	Trajectory geometry and bilateral force progression
Bilateral GRF Derivative Map	Derivative Map	Mean bilateral GRF first derivative within the bilateral force space	Rate and direction of dynamic force transitions
Bilateral GRF Occupancy Heatmap	Occupancy Heatmap	Smoothed and maximum-normalized two-dimensional histogram representing the occupancy of bilateral GRF states	Distribution and relative occupancy of bilateral GRF states

**Table 3:** Model and training characteristics of the two MobileNetV2 configurations in this study.

Parameter	MobileNetV2 (α = 0.35)	MobileNetV2 (α = 1.4)
Input image size	224 × 224 × 3	224 × 224 × 3
Feature-extractor parameters	410,208	4,363,712
Feature-vector dimension	1280	1792
Classification-layer parameters [Dense(4)]	5124	7172
Total parameters	415,332	4,370,884
Trainable parameters, Stage 1 (retraining)	5124	7172
Trainable parameters, Stage 2 (fine-tuning)	387,172	4,275,012

**Table 4:** Distribution of gait cycles among the diagnostic classes and dataset subsets.

Dataset Subset	Total	CONTROL	ALS	HUNT	PARK
Complete dataset	15,056	4076 (27.1%)	2451 (16.3%)	4845 (32.2%)	3684 (24.5%)
Training (70%)	10,539	2853	1716	3391	2579
Validation (15%)	2258	611	368	727	552
Test (15%)	2259	612	367	727	553

**Table 5:** Classification performance of the retrained MobileNetV2 models on the test subset for four-class discrimination among CONTROL, ALS, HUNT, and PARK.

Input Image	Retrained MobileNetV2	Diagnostic Class	Precision (%)	Recall (%)	Specificity (%)	F1-Score (%)	AUC	Accuracy (OvR) (%)
Trajectory Map	α = 0.35	CONTROL	91.4	91.99	96.78	91.69	0.9835	95.48
		ALS	87.5	91.55	97.46	89.48	0.9866	96.50
		HUNT	87.64	87.76	94.13	87.7	0.9673	92.08
		PARK	91.9	88.25	97.48	90.04	0.9782	95.22
	α = 1.4	CONTROL	92.74	91.83	97.33	92.28	0.9866	95.84
		ALS	83.02	95.91	96.19	89.0	0.9916	96.15
		HUNT	91.11	84.59	96.08	87.73	0.9751	92.39
		PARK	90.61	90.78	96.95	90.7	0.9838	95.44
Derivative Map	α = 0.35	CONTROL	92.31	92.16	97.15	92.23	0.9902	95.79
		ALS	94.3	92.64	98.1	91.52	0.9922	97.21
		HUNT	86.95	89.82	93.6	88.36	0.9756	92.39
		PARK	91.94	86.62	97.54	89.20	0.9808	94.86
	α = 1.4	CONTROL	95.94	88.89	98.6	92.28	0.9838	95.97
		ALS	89.6	91.55	97.94	90.57	0.9906	96.90
		HUNT	84.58	93.54	91.91	88.83	0.9777	92.43
		PARK	93.96	87.16	98.18	90.43	0.9857	95.48
Occupancy Heatmap	α = 0.35	CONTROL	91.01	95.92	96.48	93.4	0.9910	96.33
		ALS	91.92	89.92	98.47	90.91	0.9914	97.08
		HUNT	90.86	88.86	95.76	89.85	0.9792	93.54
		PARK	91.54	90.05	97.30	90.79	0.9827	95.53
	α = 1.4	CONTROL	95.8	93.14	98.48	94.45	0.9931	97.03
		ALS	94.51	93.73	98.94	94.12	0.9940	98.10
		HUNT	90.19	92.30	95.23	91.23	0.9838	94.29
		PARK	91.19	91.68	97.13	91.43	0.9873	95.79

**Table 6:** Classification performance of the fine-tuned MobileNetV2 models on the test subset for four-class discrimination among CONTROL, ALS, HUNT, and PARK.

Input Image	Fine-Tuned MobileNetV2	Diagnostic Class	Precision (%)	Recall (%)	Specificity (%)	F1-Score (%)	AUC	Accuracy (OvR) (%)
Trajectory Map	α = 0.35	CONTROL	97.19	96.08	98.97	96.63	0.9952	98.19
		ALS	96.94	94.82	99.42	95.87	0.9959	98.67
		HUNT	92.64	95.19	96.41	93.89	0.9900	96.02
		PARK	94.89	94.03	98.36	94.46	0.9940	97.30
	α = 1.4	CONTROL	98.18	96.73	99.33	97.45	0.9953	98.63
		ALS	96.47	96.73	99.31	96.60	0.9955	98.89
		HUNT	95.05	95.19	97.65	95.12	0.9933	96.86
		PARK	95.36	96.56	98.48	95.96	0.9959	98.01
Derivative Map	α = 0.35	CONTROL	96.41	96.41	98.66	96.41	0.9961	98.05
		ALS	96.42	95.37	99.31	95.89	0.9969	98.67
		HUNT	94.12	94.64	97.19	94.38	0.9906	96.37
		PARK	94.76	94.76	98.30	94.76	0.9936	97.43
	α = 1.4	CONTROL	96.21	95.42	98.60	95.82	0.9934	97.74
		ALS	95.64	95.64	99.15	95.64	0.9968	98.58
		HUNT	93.41	95.60	96.80	94.49	0.993	96.41
		PARK	97.04	94.94	99.06	95.98	0.9956	98.05
Occupancy Heatmap	α = 0.35	CONTROL	98.17	96.57	99.33	97.36	0.9953	98.58
		ALS	95.84	94.28	99.21	95.05	0.9951	98.41
		HUNT	93.69	96.01	96.93	94.84	0.9933	96.64
		PARK	95.46	95.12	98.53	95.29	0.9948	97.70
	α = 1.4	CONTROL	97.22	97.06	98.97	97.14	0.9979	98.45
		ALS	97.25	96.19	99.47	96.71	0.9979	98.94
		HUNT	94.74	96.70	97.45	95.71	0.9953	97.21
		PARK	97.24	95.48	99.12	96.35	0.9971	98.23

**Table 7:** Deployment performance of the retrained and fine-tuned MobileNetV2 models using the Trajectory Map, Derivative Map, and Occupancy Heatmap representations. Results are reported for width multipliers α = 0.35 and α = 1.4 using the original TensorFlow/Keras models on the development workstation and the standard TFLite and TFLite-DRQ models on the Raspberry Pi 4. Inference latency is reported as mean ± standard deviation across all gait-cycle images in the test subset. Estimated energy per inference was calculated for the Raspberry Pi 4 using the incremental board-level power of 2.10 W and the mean inference latency according to Equation (11). The dash (—) indicates that energy consumption was not evaluated for the workstation; DRQ: dynamic-range quantization.

Model	Setting	Deployment Platform	Model Size (kB)	Inference Latency (ms)	Estimated Incremental Energy per Inference (mJ)	Accuracy (Four-Class) (%)
**Trajectory Map**						
Retrained MobileNetV2	α = 0.35	Workstation (TF)	1968	1.92 ± 0.018	—	89.64
		Raspberry Pi 4 (TFLite)	1580	46.2 ± 2.0	97.0	89.69
		Raspberry Pi 4 (TFLite-DRQ)	595	45.4 ± 1.5	95.3	86.81
	α = 1.4	Workstation (TF)	17,437	2.27 ± 0.021	—	89.91
		Raspberry Pi 4 (TFLite)	16,833	349.3 ± 40.3	733.5	89.86
		Raspberry Pi 4 (TFLite-DRQ)	4780	330.9 ± 24.3	694.9	87.30
Fine-tuned MobileNetV2	α = 0.35	Workstation (TF)	1968	1.94 ± 0.018	—	95.09
		Raspberry Pi 4 (TFLite)	1580	45.9 ± 1.8	96.6	95.09
		Raspberry Pi 4 (TFLite-DRQ)	595	45.9 ± 1.6	96.4	94.60
	α = 1.4	Workstation (TF)	17,437	2.26 ± 0.022	—	96.19
		Raspberry Pi 4 (TFLite)	16,833	373.9 ± 5.9	785.2	96.19
		Raspberry Pi 4 (TFLite-DRQ)	4780	341.8 ± 2.8	717.8	95.57
**Derivative Map**						
Retrained MobileNetV2	α = 0.35	Workstation (TF)	1968	1.94 ± 0.016	—	90.13
		Raspberry Pi 4 (TFLite)	1580	45.7 ± 1.5	96.0	90.13
		Raspberry Pi 4 (TFLite-DRQ)	595	45.1 ± 1.5	94.7	89.11
	α = 1.4	Workstation (TF)	17,437	2.31 ± 0.019	—	90.39
		Raspberry Pi 4 (TFLite)	16,833	368.7 ± 2.5	774.3	90.31
		Raspberry Pi 4 (TFLite-DRQ)	4780	342.6 ± 2.5	719.5	85.88
Fine-tuned MobileNetV2	α = 0.35	Workstation (TF)	1968	2.00 ± 0.017	—	95.26
		Raspberry Pi 4 (TFLite)	1580	44.6 ± 7.0	93.7	95.26
		Raspberry Pi 4 (TFLite-DRQ)	595	42.2 ± 7.7	88.6	94.95
	α = 1.4	Workstation (TF)	17,437	2.46 ± 0.021	—	95.40
		Raspberry Pi 4 (TFLite)	16,833	349.2 ± 45.8	733.3	95.40
		Raspberry Pi 4 (TFLite-DRQ)	4780	323.7 ± 38.4	679.8	93.58
**Occupancy Heatmap**						
Retrained MobileNetV2	α = 0.35	Workstation (TF)	1968	1.90 ± 0.017	—	91.24
		Raspberry Pi 4 (TFLite)	1580	45.9 ± 1.7	96.4	91.24
		Raspberry Pi 4 (TFLite-DRQ)	595	45.0 ± 1.6	94.5	90.00
	α = 1.4	Workstation (TF)	17,437	2.27 ± 0.021	—	92.61
		Raspberry Pi 4 (TFLite)	16,833	367.0 ± 4.4	770.7	92.74
		Raspberry Pi 4 (TFLite-DRQ)	4780	338.2 ± 3.9	710.2	88.80
Fine-tuned MobileNetV2	α = 0.35	Workstation (TF)	1968	1.90 ± 0.019	—	95.66
		Raspberry Pi 4 (TFLite)	1580	45.2 ± 1.2	94.9	95.66
		Raspberry Pi 4 (TFLite-DRQ)	595	44.5 ± 1.0	93.5	95.35
	α = 1.4	Workstation (TF)	17,437	2.32 ± 0.023	—	96.41
		Raspberry Pi 4 (TFLite)	16,833	369.6 ± 2.0	776.2	96.41
		Raspberry Pi 4 (TFLite-DRQ)	4780	343.0 ± 2.0	720.3	96.19

**Table 8:** Comparison of the proposed MobileNetV2-based approach with previously published multiclass neurodegenerative disease gait-classification methods evaluated using the Gait in Neurodegenerative Disease Database [58].

Study	Input/Classifier	Test Type	Precision %	Recall %	F1-Score %	Accuracy %
This study	Trajectory Map/ MobileNetV2 (α = 0.35–1.4)	Patient-dependent training/validation/test: 70%/15%/15%	92.6–98.2	94.0–96.7	93.9–97.5	96.0–98.9 * 95.1–96.2 ^#^
	Derivative Map/ MobileNetV2 (α = 0.35–1.4)	Patient-dependent training/validation/test: 70%/15%/15%	93.4–97.0	94.6–96.4	94.4–96.4	96.4–98.7 * 95.3–95.4 ^#^
	Occupancy Heatmap/ MobileNetV2 (α = 0.35–1.4)	Patient-dependent training/validation/test: 70%/15%/15%	93.7–98.2	94.3–97.1	94.8–97.4	96.6–98.9 * 95.7–96.4 ^#^
Mengarelli et al., 2022 [25]	GRF gait cycles: amplitudes (statistics and integrals), intervals, slopes, sign change, variance, temporal moments, autoregressive coefficients/ KNN, SVM	Patient-dependent training/test: 70%/30%	90.0–93.7	86.0–91.9	89.2–91.9	93.6–94.8 * 89.5–93.7 ^#^
Nam Nguyen et al., 2020 [31]	GRF signal, first and second derivatives (statistical and entropy features, 10–60 s)/ KNN, SVM	Patient-dependent 10-fold CV	NA	NA	NA	94.4–99.8
Ozeloglu et al., 2024 [35]	Detrended fluctuation analysis, dynamic time warping and autocorrelation features of GRF (10 s)/SVM	NA	NA	NA	NA	93.5 ^#^
Saadeh et al., 2017 [56]	Stride time and fluctuation, autocorrelation decay factor (300 s)/DT	Patient-dependent test: 100%	NA	90.8	NA	NA
Erdaş et al., 2023 [22]	Swing, stance, double support intervals/MLP, RF, XT, KNN	Patient-dependent 10-fold CV	58.4–67.9	55.6–64.1	55.0–64.3	55.9–64.4 ^#^
	CNN		69.1	68.2	67.8	68.1 ^#^
Dutta et al., 2013 [36]	Left-to-right foot correlations of stance and swing intervals (300 s)/pre-trained Elman’s recurrent NNs	Patient-independent training/test: 50%/50%	86.3–86.8	84.0–86.8	85.1–86.8	84.0–86.8 ^#^ 83.9–87.1 *
Modaresnia et al., 2024 [38]	Left foot GRF signal (30 s)/BiLSTM	Patient-dependent 5-fold CV	70.9	70.0	70.2	70.4
	Right foot GRF signal (30 s)/BiLSTM		75.9	75.2	75.0	75.1
	Left + Right foot GRF signal (30 s)/BiLSTM		75.0	74.6	73.4	74.3
	GRF spectrogram (30 s)/AlexNet, GoogleNet, VGG16		99.3–99.9	99.3–99.9	99.3–99.9	99.2–99.9
Zeng et al., 2026 [39]	GRF signal (gait cycles)/ Dual-Branch Attention-Enhanced Residual Network	Patient-dependent training/test: 80%/20%	99.7	99.6	99.7	99.6 ^#^
		5-fold CV	99.5	99.5	99.5	99.5 ^#^
		LOO (gait cycle level)	98.3	98.3	98.3	98.3 ^#^
		Patient-independent holdout (patient level)	98.2	98.1	98.1	98.1 ^#^
Erdaş et al., 2024 [40]	QR codes from swing, stance, double support intervals/CNN	Patient-dependent 10-fold CV	90.6	80.1	84.0	84.7 *
Setiawan et al., 2021 [41]	Spectrogram-based analysis of GRF (10–60 s)/AlexNet + SVM	Patient-dependent LOO or 5-fold CV				
	CWT spectrograms		NA	97.2–97.3	NA	98.4–98.5 *
	STFT spectrograms		NA	92.6–93.1	NA	96.3–96.4 *
	WSST spectrograms		NA	89.2–90.8	NA	94.6–94.9 *
Torghabeh et al., 2024 [42]	Wavelet coherence images of GRF signals of left vs. right foot (5–10 s)/	Patient-dependent 5-fold CV				
	AlexNet		95.7–99.3	96.0–99.2	95.8–99.2	95.9–99.2
	GoogLeNet		96.3–96.7	96.7–98.8	96.4–96.7	96.5–96.8
	SqueezeNet		92.6–94.7	92.6–95.1	92.6–94.8	92.7–95.0
Lin et al., 2020 [44]	GRF recurrence plots (10 s)/ AlexNet + SVN	Patient-dependent LOO CV	NA	93.9–95.5	NA	97.1–97.9 *

Note: *: indicates class-specific one-vs-rest accuracy; ^#^ indicates overall four-class classification accuracy. Where neither symbol is provided, the type of reported accuracy could not be determined unambiguously from the original publication. In such cases, the accuracy values are reproduced as reported without assigning an unverified metric definition. Patient-dependent evaluation refers to sample-wise partitioning in which data from the same subject may appear in both training and test subsets, whereas patient-independent evaluation refers to subject-wise partitioning in which test subjects are excluded from training. Test performance values are reported as presented in the original publications, except for Dutta et al., 2013 [36] for which precision, recall, and F1-score were calculated from the information provided in the publication. Because the studies differ in signal representations, input durations, classification methods, evaluation protocols, and metric definitions, the reported performance values should not be interpreted as directly comparable. Abbreviations: BiLSTM: bidirectional long short-term memory; CNN: convolutional neural network; CV: cross-validation; CWT: continuous wavelet transform; DT: decision tree; KNN: k-nearest neighbors; LOO: leave-one-out; MLP: multilayer perceptron; NA: not available; RF: random forest; STFT: short-time Fourier transform; SVM: support vector machine; WSST: wavelet synchrosqueezed transform; XT: extremely randomized trees.

**Table 9:** Comparison of model size and inference time of various models for gait analysis implemented on embedded computing platforms.

Study	Task	Input (Size)	Test Platform	Model	Model Size	Inference Time
This study	NDD classification in four classes: CONTROL, ALS, HUNT, PARK	Trajectory Map Derivative Map Occupancy Heatmap	Raspberry Pi 4	MobileNetV2 (32-bit)	1.6–16.8 MB 1.6–16.8 MB 1.6–16.8 MB	45.9–373.9 ms 42.2–368.7 ms 45.2–369.6 ms
This study	NDD classification in four classes: CONTROL, ALS, HUNT, PARK	Trajectory Map Derivative Map Occupancy Heatmap	Raspberry Pi 4	MobileNetV2 (8-bit)	0.6–4.8 MB 0.6–4.8 MB 0.6–4.8 MB	45.4–341.8 ms 44.6–342.6 ms 44.5–343.0 ms
Gokul et al., 2020 [48]	FoG detection in PARK	Accelerometer features (2 s)	Arduino Mega (ATMega2560) microcontroller	ProtoNN (KNN-based)	2.92 kB	20 ms *
Al-Adhaileh et al., 2025 [51]	FoG detection in PARK	Accelerometer and gyroscope signals (2 s)	Raspberry Pi 4	CNNs with BiLSTM (8-bit quantized)	2.9 MB	211 ms
Hou et al., 2023 [49]	FoG detection in PARK	Accelerometer, EMG, EEG signals (3 s)	Arm Cortex M4	Depthwise CNN (8-bit quantized)	830 kB	2300 ms
Slemenšek et al., 2024 [50]	FoG detection in PARK	Accelerometer, muscle activity, gyroscope signals (1 s)	Arduino Portenta H7 microcontroller	CNN + RNN (32-bit)	NA	261 ms

Note: Inference time refers to model/classifier execution time where reported separately from preprocessing. (*) For Gokul et al., 2020 [48], the reported value of 20 ms corresponds to the ProtoNN classification module; the authors reported a minimum total ML-subsystem latency of 44.5 ms when feature extraction was included. Because the studies differ in classification task, sensing modality, input duration, hardware platform, and latency definition, the reported inference times should not be interpreted as directly comparable. Abbreviations: ALS: amyotrophic lateral sclerosis; BiLSTM: bidirectional long short-term memory; CNN: convolutional neural network; EEG: electroencephalography; EMG: electromyography; FoG: freezing of gait; HUNT: Huntington’s disease; KNN: K-nearest neighbors; NA: not available; NDD: neurodegenerative disease; PARK: Parkinson’s disease; RNN: recurrent neural network.

## Data Availability

The Gait in Neurodegenerative Disease Database is available at https://www.physionet.org/content/gaitndd/1.0.0/ (accessed on 24 July 2026).

## Supplementary Materials

The following supporting information can be downloaded at: https://www.mdpi.com/article/10.3390/s26217010/s1, File S1: Supplementary Materials S1. Section S1: Paired statistical comparisons of MobileNetV2 configurations; Section S2: Confusion matrices and overall classification metrics; Section S3: Sex- and age-stratified classification performance; Section S4: Subject-wise gait-cycle classification performance. Table S1: Paired comparisons of overall four-class classification accuracy between MobileNetV2 configurations; Table S2: Confusion matrices, macro-F1 scores, and balanced accuracy for the retrained MobileNetV2 models; Table S3: Confusion matrices, macro-F1 scores, and balanced accuracy for the fine-tuned MobileNetV2 models; Table S4: Sex-stratified class-specific recall for the six fine-tuned MobileNetV2 configurations; Table S5: Age-stratified class-specific recall for the six fine-tuned MobileNetV2 configurations; Table S6: Summary statistics of subject-wise gait-cycle classification accuracy across all 12 MobileNetV2 configurations; Table S7: Subject-wise distributions of predicted gait-cycle classes and classification accuracy for the retrained MobileNetV2 model (α = 0.35); Table S8: Subject-wise distributions of predicted gait-cycle classes and classification accuracy for the retrained MobileNetV2 model (α = 1.4); Table S9: Subject-wise distributions of predicted gait-cycle classes and classification accuracy for the fine-tuned MobileNetV2 model(α = 0.35); Table S10: Subject-wise distributions of predicted gait-cycle classes and classification accuracy for the fine-tuned MobileNetV2 model (α = 1.4).

## Abbreviations

The following abbreviations are used in this manuscript:

**Abbreviation**	**Definition**
ALS	Amyotrophic Lateral Sclerosis
ANFIS	Adaptive Neuro-Fuzzy Inference System
AUC	Area Under the Receiver Operating Characteristic Curve
BiLSTM	Bidirectional Long Short-Term Memory
CI	Confidence Interval
CNN	Convolutional Neural Network
CONTROL	Healthy Control Group
DRQ	Dynamic-Range Quantization
FN	False Negative
FoG	Freezing of Gait
FP	False Positive
FPGA	Field-Programmable Gate Array
GB	Gradient Boosting
GNDD	Gait in Neurodegenerative Disease Database
GPU	Graphics Processing Unit
GRF	Ground Reaction Force
HUNT	Huntington’s Disease
IDE	Integrated Development Environment
KNN	K-Nearest Neighbors
LDA	Linear Discriminant Analysis
LR	Linear Regression
MB	Megabyte
ML	Machine Learning
MLR	Multiclass Logistic Regression
NB	Naive Bayes
NDD	Neurodegenerative Disease
NN	Neural Network
OS	Operating System
OvR	One-vs-Rest
PARK	Parkinson’s Disease
RAM	Random Access Memory
RGB	Red, Green, Blue
ROC	Receiver Operating Characteristic
SDRAM	Synchronous Dynamic Random Access Memory
SVM	Support Vector Machine
TFLite	TensorFlow Lite
TN	True Negative
TP	True Positive
