# Supplementary material for: MobileNetV2 for Multiclass Neurodegenerative Gait Analysis Using Bilateral Ground Reaction Force Images: From Transfer Learning to Raspberry Pi 4 Deployment
Source: Sensors (Basel). 2026 Nov 5;26(21):7010. doi: 10.3390/s26217010 (PMC13766332; doi:10.3390/s26217010)
Supplement: Supplementary file 1 [file sensors-26-07010-s001.zip › Supplementary Material S1.pdf]

sensors

MDPI

Article

MobileNetV2 for Multiclass Neurodegenerative Gait Analysis Using Bilateral Ground Reaction Force Images: From Transfer Learning to Raspberry Pi 4 Deployment (Supplementary Material 1)

Vessela Krasteva, Todor Stoyanov, and Irena Jekova *

Institute of Biophysics and Biomedical Engineering, Bulgarian Academy of Sciences, Acad. G. Bonchev Str. Bl. 105, 1113 Sofia, Bulgaria; vessika@biomed.bas.bg (VK); todor@biomed.bas.bg (TS)
* Correspondence: irena@biomed.bas.bg

S1. Paired Statistical Comparisons of MobileNetV2 Configurations

Table S1 presents the results of 24 paired comparisons of overall four-class classification accuracy between MobileNetV2 configurations, grouped according to the experimental factor varied: Ground reaction force (GRF) image representation (12 comparisons), network width multiplier (6 comparisons), and training strategy (6 comparisons). In each comparison, predictions from the same test gait cycles were evaluated while the remaining configuration factors were held constant.

Table S1. Paired comparisons of overall four-class classification accuracy between MobileNetV2 configurations on the test subset. Accuracy differences (Δ accuracy) are calculated as Model A − Model B and expressed in percentage points (pp). The corresponding percentile-based 95% confidence intervals (CIs) were obtained from 10,000 subject-clustered bootstrap replicates and were not adjusted for multiple comparisons. "Only A correct" and "Only B correct" indicate the numbers of test gait cycles correctly classified exclusively by Model A or Model B, respectively. Exact McNemar tests were performed on paired gait-cycle predictions, with p-values adjusted using the Holm procedure separately within each comparison family. These tests were considered exploratory because they did not account for within-subject dependence. Bold Holm-adjusted p-values indicate $p < 0.05$. Trajectory, Derivative, and Occupancy refer to the corresponding bilateral GRF image representations defined in Section 2.2 of the main manuscript. Further details of the statistical procedures are provided in Section 2.3.3.3 of the main manuscript.

Comparison	Model A	Model B	Δ Accuracy (95% CI), pp	Only A correct	Only B correct	Exact p	Holm-adjusted p
**GRF image representation comparisons (n = 12)**							
1	Retrained, Trajectory, $\alpha = 0.35$	Retrained, Derivative, $\alpha = 0.35$	−0.49 (−2.26 to +1.17)	141	152	0.5592	1.00
2	Retrained, Trajectory, $\alpha = 0.35$	Retrained, Occupancy, $\alpha = 0.35$	−1.59 (−3.37 to +0.22)	120	156	0.0349	0.3145
3	Retrained, Derivative, $\alpha = 0.35$	Retrained, Occupancy, $\alpha = 0.35$	−1.11 (−2.81 to +0.60)	136	161	0.1636	1.00

 https://doi.org/10.3390/s26217010

4	Retrained, Trajectory, α = 1.4	Retrained, Derivative, α = 1.4	−0.49 (−2.63 to +1.43)	137	148	0.5537	1.00
5	Retrained, Trajectory, α = 1.4	Retrained, Occupancy, α = 1.4	−2.70 (−4.51 to −0.99)	101	162	0.0002	**0.0024**
6	Retrained, Derivative, α = 1.4	Retrained, Occupancy, α = 1.4	−2.21 (−3.73 to −0.77)	104	154	0.0022	**0.0244**
7	Fine-tuned, Trajectory, α = 0.35	Fine-tuned, Derivative, α = 0.35	−0.18 (−1.31 to +0.88)	61	65	0.7894	1.00
8	Fine-tuned, Trajectory, α = 0.35	Fine-tuned, Occupancy, α = 0.35	−0.58 (−1.66 to +0.43)	50	63	0.2589	1.00
9	Fine-tuned, Derivative, α = 0.35	Fine-tuned, Occupancy, α = 0.35	−0.40 (−1.48 to +0.67)	60	69	0.4814	1.00
10	Fine-tuned, Trajectory, α = 1.4	Fine-tuned, Derivative, α = 1.4	+0.80 (−0.22 to +1.76)	58	40	0.0854	0.6833
11	Fine-tuned, Trajectory, α = 1.4	Fine-tuned, Occupancy, α = 1.4	−0.22 (−0.96 to +0.50)	39	44	0.6609	1.00
12	Fine-tuned, Derivative, α = 1.4	Fine-tuned, Occupancy, α = 1.4	−1.02 (−1.89 to −0.14)	37	60	0.0250	0.2499
**MobileNetV2 width comparisons (n = 6)**							
13	Retrained, Trajectory, α = 1.4	Retrained, Trajectory, α = 0.35	+0.27 (−1.37 to +1.87)	134	128	0.7575	1.00
14	Retrained, Derivative, α = 1.4	Retrained, Derivative, α = 0.35	+0.27 (−1.05 to +1.52)	132	126	0.7557	1.00
15	Retrained, Occupancy, α = 1.4	Retrained, Occupancy, α = 0.35	+1.37 (−0.09 to +2.88)	132	101	0.0491	0.2457
16	Fine-tuned, Trajectory, α = 1.4	Fine-tuned, Trajectory, α = 0.35	+1.11 (+0.30 to +1.96)	58	33	0.0115	0.0687
17	Fine-tuned, Derivative, α = 1.4	Fine-tuned, Derivative, α = 0.35	+0.13 (−0.74 to +0.97)	51	48	0.8408	1.00
18	Fine-tuned, Occupancy, α = 1.4	Fine-tuned, Occupancy, α = 0.35	+0.75 (−0.14 to +1.64)	56	39	0.1002	0.4007
**Training strategy comparisons (n = 6)**							
19	Fine-tuned, Trajectory, α = 0.35	Retrained, Trajectory, α = 0.35	+5.44 (+4.13 to +6.81)	143	20	<0.0001	**<0.0001**
20	Fine-tuned, Trajectory, α = 1.4	Retrained, Trajectory, α = 1.4	+6.29 (+4.79 to +7.88)	157	15	<0.0001	**<0.0001**
21	Fine-tuned, Derivative, α = 0.35	Retrained, Derivative, α = 0.35	+5.14 (+3.80 to +6.61)	144	28	<0.0001	**<0.0001**
22	Fine-tuned, Derivative, α = 1.4	Retrained, Derivative, α = 1.4	+5.00 (+3.71 to +6.41)	131	18	<0.0001	**<0.0001**
23	Fine-tuned, Occupancy, α = 0.35	Retrained, Occupancy, α = 0.35	+4.43 (+3.34 to +5.58)	122	22	<0.0001	**<0.0001**
24	Fine-tuned, Occupancy, α = 1.4	Retrained, Occupancy, α = 1.4	+3.81 (+2.59 to +5.10)	111	25	<0.0001	**<0.0001**

S2. Confusion Matrices and Overall Classification Metrics

The confusion matrices for the retrained and fine-tuned MobileNetV2 models on the test subset are presented in Tables S2 and S3, respectively. The models perform four-class classification of gait cycles from healthy control subjects (CONTROL) and patients with amyotrophic lateral sclerosis (ALS), Huntington's disease (HUNT), and Parkinson's disease (PARK). The matrices report the numbers of gait cycles assigned to each predicted diagnostic class for each true class in the test subset. In addition to the class-specific performance metrics reported in the main manuscript, macro-averaged F1-score and balanced accuracy are included to summarize multiclass classification performance.

Macro-F1 is calculated as the unweighted mean of the F1-scores across the four diagnostic classes, whereas balanced accuracy is calculated as the unweighted mean of the corresponding class-specific recalls (Equation (1)). Both metrics assign equal weight to each diagnostic class, irrespective of the number of gait cycles belonging to that class.

$$\text{Macro-F1} = \frac{1}{4}\sum_{C=1}^{4} \text{F1}_C, \qquad \text{Balanced Accuracy} = \frac{1}{4}\sum_{C=1}^{4} \text{Recall}_\text{C}, \tag{1}$$

where

$$\text{F1}_\text{C} = \frac{2\text{TP}_\text{C}}{2\text{TP}_\text{C} + \text{FP}_\text{C} + \text{FN}_\text{C}} \times 100\ (\%), \qquad \text{Recall}_\text{C} = \frac{2\text{TP}_\text{C}}{\text{TP}_\text{C} + \text{FN}_\text{C}} \times 100(\%), \tag{2}$$

Here, TPc, FPc, and FNc denote the true positives, false positives, and false negatives for diagnostic class c, respectively.

Table S2. Confusion matrices and overall performance metrics of the retrained MobileNetV2 models on the test subset for four-class classification of CONTROL, ALS, HUNT, and PARK gait cycles. Rows represent the true diagnostic classes, and columns represent the predicted classes. Shaded cells indicate correct classifications (true positives). Macro-F1 and balanced accuracy are reported for each model configuration.

Input image	Retrained MobileNetV2	Diagnostic Class	CON-TROL	ALS	HUNT	PARK	Macro-F1 (%)	Balanced Accuracy (%)
Trajectory Map	α=0.35	CONTROL	563	5	33	11	89.73	89.89
		ALS	5	336	19	7		
		HUNT	33	31	638	25		
		PARK	15	12	38	488		
	α=1.4	CONTROL	562	6	30	14	89.93	90.78
		ALS	4	352	8	3		
		HUNT	26	51	615	35		
		PARK	14	15	22	502		
Derivative Map	α=0.35	CONTROL	564	5	32	11	90.33	90.31
		ALS	5	340	19	3		
		HUNT	23	23	653	28		
		PARK	19	8	47	479		
	α=1.4	CONTROL	544	7	50	11	90.53	90.28
		ALS	6	336	23	2		
		HUNT	9	20	680	18		
		PARK	8	12	51	482		
Occupancy Heatmap	α=0.35	CONTROL	587	3	15	7	91.24	91.19
		ALS	9	330	17	11		
		HUNT	33	20	646	28		
		PARK	16	6	33	498		
	α=1.4	CONTROL	570	2	26	14	92.81	92.71
		ALS	3	344	12	8		
		HUNT	12	17	671	27		
		PARK	10	1	35	507		

Abbreviations. CONTROL: healthy control subjects; ALS: amyotrophic lateral sclerosis; HUNT: Huntington's disease; PARK: Parkinson's disease; α: MobileNetV2 width multiplier.

Table S3. Confusion matrices and overall performance metrics of the fine-tuned MobileNetV2 models on the test subset for four-class classification of CONTROL, ALS, HUNT, and PARK gait cycles**.** Rows represent the true diagnostic classes, and columns represent the predicted classes. Shaded cells indicate correct classifications (true positives). Macro-F1 and balanced accuracy are reported for each model configuration.

Input image	Fine-tuned MobileNetV2	Diagnostic Class	CON-TROL	ALS	HUNT	PARK	Macro F1-score	Balanced Accuracy
Trajectory Map	α=0.35	CONTROL	588	1	17	6	95.21	95.03
		ALS	1	348	14	4		
		HUNT	11	6	692	18		
		PARK	5	4	24	520		
	α=1.4	CONTROL	592	2	12	6	96.28	96.30
		ALS	2	355	10	0		
		HUNT	6	9	692	20		
		PARK	3	2	14	534		
Derivative Map	α=0.35	CONTROL	590	3	11	8	95.36	95.29
		ALS	1	350	12	4		
		HUNT	13	9	688	17		
		PARK	8	1	20	524		
	α=1.4	CONTROL	584	2	21	5	95.48	95.40
		ALS	5	351	10	1		
		HUNT	12	10	695	10		
		PARK	6	4	18	525		
Occupancy Heatmap	α=0.35	CONTROL	591	2	15	4	95.64	95.49
		ALS	1	346	13	7		
		HUNT	7	8	698	14		
		PARK	3	5	19	526		
	α=1.4	CONTROL	594	0	13	5	96.48	96.36
		ALS	2	353	10	2		
		HUNT	9	7	703	8		
		PARK	6	3	16	528		

Abbreviations. CONTROL: healthy control subjects; ALS: amyotrophic lateral sclerosis; HUNT: Huntington's disease; PARK: Parkinson's disease; α: MobileNetV2 width multiplier.

S3. Sex- and Age-Stratified Classification Performance

Exploratory subgroup analyses were performed using predictions from the held-out test gait cycles described in Section 2.3.3.1 and Table 4 of the main manuscript to assess potential differences in class-specific recall according to sex and age across the six fine-tuned MobileNetV2 configurations. Sex-stratified analyses compared male and female participants within each diagnostic category (CONTROL, ALS, HUNT, and PARK). For age-stratified analyses, participants were divided into younger and older subgroups using the diagnosis-specific median age (CONTROL: 35 years; ALS: 62 years; HUNT: 44.5 years; PARK: 68 years), with participants at or below the median assigned to the younger subgroup.

Class-specific recall, defined in Equation (8) of the main manuscript, was calculated from individual gait-cycle predictions, with each gait cycle contributing equally to the corresponding subgroup estimate. To account for multiple gait cycles originating from the same participant, 95% confidence intervals (CIs) for subgroup-specific recall and between-subgroup differences were estimated using 10,000 patient-clustered bootstrap replicates. Differences were expressed in percentage points (pp) and calculated as male minus female or younger minus older, respectively. Statistical comparisons were performed using two-sided patient-level permutation tests with 10,000 permutations. The resulting p-values were adjusted for multiple comparisons using the Holm procedure across the four diagnostic categories within each model configuration.

Sex-stratified and age-stratified results are presented in Tables S4 and S5, respectively. No subgroup comparison remained statistically significant after Holm adjustment (adjusted $p > 0.05$). These findings should be interpreted cautiously because of the limited and, particularly for sex, imbalanced subgroup sizes. The absence of statistically significant differences does not establish equivalent classification performance across demographic groups.

Table S4. Sex-stratified class-specific recall for the six fine-tuned MobileNetV2 configurations across the four diagnostic categories in the test subset. Recall values are expressed as percentages with 95% confidence intervals (CIs) estimated using 10,000 patient-clustered bootstrap replicates. Differences are calculated as male minus female recall and expressed in percentage points (pp). Holm-adjusted p-values were obtained from two-sided patient-level permutation tests (10,000 permutations), with adjustment across the four diagnostic categories within each model configuration. Sample sizes (n) represent the number of individual participants. Results should be interpreted as exploratory because of small and imbalanced sex-specific subgroups.

Model / input representation	Diagnosis	Male n	Female n	Male recall % (95% CI)	Female recall % (95% CI)	Difference pp (95% CI)	Holm adjusted p-value
MobileNetV2 (α=0.35) Trajectory Map	CONTROL	2	14	96.34 (94.74–97.73)	96.04 (93.91–98.03)	0.30 (−2.70 to 3.17)	1.000
	ALS	10	3	95.54 (93.49–97.52)	92.86 (80.95–97.22)	2.68 (−2.19 to 14.24)	1.000
	HUNT	6	14	97.25 (93.93–99.60)	94.07 (90.29–97.14)	3.19 (−1.26 to 7.79)	1.000
	PARK	10	5	93.15 (88.29–96.56)	95.74 (93.41–97.41)	−2.59 (−7.44 to 1.58)	1.000
MobileNetV2 (α=1.4) Trajectory	CONTROL	2	14	97.56 (94.74–100.00)	96.60 (94.70–98.26)	0.96 (−3.05 to 4.59)	1.000
	ALS	10	3	97.40	94.90	2.50	0.678

Map				(96.03–98.81)	(92.68–97.22)	(−0.21 to 5.09)	
	HUNT	6	14	95.29 (92.86–97.45)	95.13 (92.16–97.60)	0.17 (−3.34 to 3.78)	1.000
	PARK	10	5	96.16 (92.88–98.65)	97.34 (95.14–99.48)	−1.18 (−5.00 to 2.32)	1.000
MobileNetV2 (α=0.35) Derivative Map	CON-TROL	2	14	96.34 (94.74–97.73)	96.42 (94.39–98.19)	−0.07 (−2.96 to 2.65)	1.000
	ALS	10	3	95.54 (92.89–97.46)	94.90 (85.71–97.56)	0.64 (−3.63 to 9.45)	1.000
	HUNT	6	14	95.29 (93.16–97.69)	94.28 (90.97–96.90)	1.01 (−2.43 to 4.86)	1.000
	PARK	10	5	93.97 (90.83–96.77)	96.28 (93.65–98.45)	−2.30 (−6.20 to 1.58)	1.000
MobileNetV2 (α=1.4) Derivative Map	CON-TROL	2	14	92.68 (86.84–97.73)	95.85 (93.64–97.98)	−3.17 (−10.48 to 3.29)	1.000
	ALS	10	3	96.28 (94.09–98.29)	93.88 (85.71–97.22)	2.40 (−1.55 to 10.12)	1.000
	HUNT	6	14	95.29 (92.26–98.63)	95.76 (93.51–97.60)	−0.47 (−3.94 to 3.36)	1.000
	PARK	10	5	94.79 (92.26–97.10)	95.21 (93.44–96.94)	−0.42 (−3.53 to 2.47)	1.000
MobileNetV2 (α=0.35) Occupancy Heatmap	CON-TROL	2	14	95.12 (92.11–97.73)	96.79 (94.83–98.59)	−1.67 (−5.94 to 2.16)	1.000
	ALS	10	3	94.05 (91.35–96.20)	94.90 (85.71–97.56)	−0.85 (−5.28 to 7.82)	1.000
	HUNT	6	14	95.69 (91.87–99.18)	96.19 (94.27–97.91)	−0.50 (−4.73 to 3.41)	1.000
	PARK	10	5	94.25 (91.34–96.72)	96.81 (93.75–99.47)	−2.56 (−6.47 to 1.31)	1.000
MobileNetV2 (α=1.4) Occupancy Heatmap	CON-TROL	2	14	97.56 (97.37–97.73)	96.98 (95.31–98.53)	0.58 (−1.05 to 2.25)	1.000
	ALS	10	3	95.91 (93.94–97.86)	96.94 (95.24–97.56)	−1.03 (−3.10 to 1.33)	1.000
	HUNT	6	14	98.43 (97.08–99.61)	95.76 (92.89–98.05)	2.67 (0.03 to 5.78)	0.827
	PARK	10	5	94.52 (91.32–97.38)	97.34 (95.58–98.91)	−2.82 (−6.30 to 0.50)	0.852

Table S5. Age-stratified class-specific recall for the six fine-tuned MobileNetV2 configurations across the four diagnostic categories in the test subset. Participants were divided into younger and older subgroups according to the median age within each diagnostic category (CONTROL: 35 years; ALS: 62 years; HUNT: 44.5 years; PARK: 68 years). Participants aged at or below the diagnosis-specific median were assigned to the younger subgroup, and those above the median to the older subgroup. Recall values are expressed as percentages with 95% confidence intervals (CIs) estimated using 10,000 patient-clustered bootstrap replicates. Differences are calculated as younger minus older recall and expressed in percentage points (pp). Holm-adjusted p-values were obtained from two-sided patient-level permutation tests (10,000 permutations), with adjustment across the four diagnostic categories within each model configuration. Sample sizes (n) represent the number of individual participants. Results should be interpreted as exploratory because of the limited subgroup sizes.

Model / input representation	Diagnosis	Median age (years)	Younger n	Older n	Younger recall % (95% CI)	Older recall % (95% CI)	Difference pp (95% CI)	Holm adjusted p-value
MobileNetV2 (α=0.35) Trajectory Map	CONTROL	35	8	8	95.11 (92.38–97.70)	97.05 (94.67–99.01)	−1.94 (−5.34 to 1.58)	0.685
	ALS	62	7	6	96.45 (95.02–98.18)	92.94 (88.00–96.53)	3.51 (−0.47 to 8.79)	0.473
	HUNT	44.5	10	10	94.93 (90.06–98.26)	95.45 (92.40–98.07)	−0.52 (−5.98 to 4.03)	0.862
	PARK	68	8	7	91.55 (86.08–95.67)	96.89 (96.05–97.44)	−5.33 (−10.80 to −1.19)	0.202
MobileNetV2 (α=1.4) Trajectory Map	CONTROL	35	8	8	95.44 (92.81–97.74)	98.03 (96.20–99.60)	−2.59 (−5.65 to 0.35)	0.568
	ALS	62	7	6	97.46 (96.15–98.65)	95.88 (93.97–98.58)	1.58 (−1.37 to 3.87)	0.741
	HUNT	44.5	10	10	94.40 (90.83–97.24)	96.02 (93.88–97.84)	−1.62 (−5.64 to 1.99)	0.741
	PARK	68	8	7	95.27 (91.21–98.39)	98.05 (96.75–99.21)	−2.78 (−7.05 to 0.61)	0.741
MobileNetV2 (α=0.35) Derivative Map	CONTROL	35	8	8	95.44 (92.56–97.99)	97.38 (95.64–98.99)	−1.94 (−5.27 to 1.12)	1.000
	ALS	62	7	6	94.92 (91.19–97.01)	95.88 (92.07–98.73)	−0.96 (−5.69 to 3.41)	1.000
	HUNT	44.5	10	10	94.93 (92.23–96.98)	94.32 (90.75–97.54)	0.62 (−3.55 to 4.77)	1.000
	PARK	68	8	7	93.92 (90.49–96.87)	95.72 (92.42–98.01)	−1.80 (−6.14 to 2.63)	1.000
MobileNetV2 (α=1.4) Derivative Map	CONTROL	35	8	8	93.49 (90.13–96.82)	97.38 (95.18–99.35)	−3.89 (−7.85 to 0.10)	0.404
	ALS	62	7	6	95.94 (93.16–98.16)	95.29 (91.84–98.16)	0.64 (−3.46 to 4.70)	1.000
	HUNT	44.5	10	10	95.20 (92.22–97.65)	96.02 (94.10–97.98)	−0.82 (−4.36 to 2.35)	1.000
	PARK	68	8	7	93.92	96.11	−2.19	0.719

					(91.46–95.85)	(93.41–98.41)	(−5.60 to 1.09)	
MobileNetV2 (α=0.35) Occupancy Heatmap	CON-TROL	35	8	8	96.09 (93.46–98.67)	97.05 (94.70–98.99)	−0.96 (−4.33 to 2.51)	1.000
	ALS	62	7	6	94.92 (94.05–95.96)	93.53 (87.94–97.46)	1.39 (−2.72 to 7.06)	1.000
	HUNT	44.5	10	10	95.73 (93.05–98.10)	96.31 (93.79–98.42)	−0.57 (−3.98 to 2.93)	1.000
	PARK	68	8	7	93.92 (90.27–96.97)	96.50 (94.44–98.44)	−2.58 (−6.80 to 1.13)	1.000
MobileNetV2 (α=1.4) Occupancy Heatmap	CON-TROL	35	8	8	96.42 (94.32–98.33)	97.70 (95.82–99.35)	−1.29 (−3.99 to 1.39)	1.000
	ALS	62	7	6	95.94 (93.84–97.49)	96.47 (93.83–98.76)	−0.53 (−3.74 to 2.49)	1.000
	HUNT	44.5	10	10	96.00 (92.19–98.68)	97.44 (95.66–98.97)	−1.44 (−5.51 to 1.76)	1.000
	PARK	68	8	7	93.92 (90.57–96.43)	97.28 (94.31–99.60)	−3.36 (−7.66 to 0.55)	0.621

S4. Subject-wise Gait-Cycle Classification Performance

To additionally characterize the variability in classification performance among individual subjects, subject-wise gait-cycle classification accuracy was evaluated using the test subset described in Section 2.3.3.1 and Table 4 of the main manuscript. For each subject and MobileNetV2 configuration, accuracy was calculated as the percentage of that subject's test gait cycles correctly assigned to the corresponding diagnostic class. Summary statistics were calculated across all 64 subjects, with each subject contributing equally, irrespective of the number of test gait cycles.

Table S6 summarizes the distribution of subject-wise classification accuracy for the six retrained and six fine-tuned MobileNetV2 configurations, reporting the mean and standard deviation (SD), median, and minimum–maximum values. The mean subject-wise accuracy ranged from 89.34% to 92.32% for the retrained models and from 94.88% to 96.25% for the fine-tuned models. Fine-tuning increased the mean subject-wise accuracy and reduced its standard deviation across all six matched configurations. Nevertheless, the minimum accuracy values indicate that classification performance varied among individual subjects, even for the fine-tuned models.

The detailed distribution of gait-cycle predictions for individual subjects in the test subset is presented in Tables S7–S10. For each subject, these tables report the number of test gait cycles assigned to each of the four diagnostic classes and the percentage of correctly classified gait cycles, separately for the Trajectory Map, Derivative Map, and Occupancy Heatmap representations. Tables S7 and S8 correspond to the retrained MobileNetV2 models with width multipliers $\alpha = 0.35$ and $\alpha = 1.4$, respectively, whereas Tables S9 and S10 present the corresponding results for the fine-tuned models.

These analyses complement the overall and class-specific classification results reported in Section 3.1 of the main manuscript by describing the variability in gait-cycle classification performance across individual subjects. Because gait cycles from the same subject could occur in the training, validation, and test subsets, the reported subject-wise accuracies do not represent subject-independent evaluation or classification performance in previously unseen subjects.

Table S6. Summary statistics of subject-wise gait-cycle classification accuracy for the six retrained and six fine-tuned MobileNetV2 configurations on the test subset (64 subjects). Accuracy is reported as mean ± standard deviation (SD), median with the first and third quartiles (Q1–Q3), and minimum–maximum values. All statistics were calculated from individual subject-wise accuracies, with each subject contributing equally, irrespective of the number of test gait cycles.

Training strategy	α	Representation	Mean ± SD (%)	Median (Q1-Q3) (%)	Min–max (%)
Retrained	0.35	Trajectory Map	89.34 ± 9.19	92.57 (84.47–96.14)	61.76–100
		Derivative Map	90.01 ± 8.25	92.55 (85.08–96.99)	66.67–100
		Occupancy Heatmap	90.61 ± 8.10	92.76 (86.09–97.16)	66.67–100
	1.4	Trajectory Map	89.76 ± 10.65	92.00 (85.93–97.24)	47.06–100
		Derivative Map	89.97 ± 8.60	92.64 (86.56–94.66)	51.85–100
		Occupancy Heatmap	92.32 ± 7.87	94.52 (89.40–97.22)	61.90–100
Fine-tuned	0.35	Trajectory Map	94.88 ± 5.47	96.93 (94.08–97.73)	76.47–100
		Derivative Map	95.02 ± 4.80	96.87 (93.16–97.67)	80.95–100
		Occupancy Heatmap	95.42 ± 4.19	96.88 (92.91–98.58)	83.33–100
	1.4	Trajectory Map	96.12 ± 4.19	97.26 (94.36–100)	81.48–100
		Derivative Map	95.26 ± 4.41	95.45 (92.66–100)	80.95–100
		Occupancy Heatmap	96.25 ± 4.37	97.34 (94.97–100)	76.19–100

Table S7. Subject-wise distribution of predicted gait-cycle classes and classification accuracy (%) for the retrained MobileNetV2 model (α = 0.35) on the test subset, using the Trajectory Map, Derivative Map, and Occupancy Heatmap representations. Abbreviations. C: CONTROL (healthy control subjects); A: ALS (amyotrophic lateral sclerosis); H: HUNT (Huntington's disease); P: PARK (Parkinson's disease); n: number of gait cycles; Acc: Accuracy - percentage of correctly classified gait cycles for the corresponding subject; α: MobileNetV2 width multiplier.

GNDD Database		Trajectory Map					Derivative Map					Occupancy Heatmap				
Patient ID	Testset Gait cycles, n	C n	A, n	H, n	P, n	Acc (%)	C, n	A, n	H, n	P, n	Acc (%)	C, n	A, n	H, n	P, n	Acc (%)
**als1**	26	0	22	2	2	84.62	0	24	2	0	92.31	0	26	0	0	100
**als10**	36	0	35	1	0	97.22	0	34	2	0	94.44	0	35	1	0	97.22
**als11**	31	0	29	2	0	93.55	0	27	3	1	87.10	1	30	0	0	96.77
**als12**	20	0	20	0	0	100	0	20	0	0	100	0	17	3	0	85.00
**als13**	22	0	21	1	0	95.45	0	21	1	0	95.45	1	17	2	2	77.27
**als2**	36	1	34	0	1	94.44	1	35	0	0	97.22	2	33	0	1	91.67
**als3**	41	1	39	1	0	95.12	1	37	3	0	90.24	1	39	1	0	95.12
**als4**	21	2	16	2	1	76.19	3	17	1	0	80.95	1	17	1	2	80.95
**als5**	17	0	14	3	0	82.35	0	14	3	0	82.35	0	16	1	0	94.12
**als6**	21	0	20	1	0	95.24	0	20	0	1	95.24	1	18	1	1	85.71
**als7**	24	0	19	3	2	79.17	0	20	3	1	83.33	0	16	5	3	66.67
**als8**	33	1	31	0	1	93.94	0	33	0	0	100	1	29	1	2	87.88
**als9**	39	0	36	3	0	92.31	0	38	1	0	97.44	1	37	1	0	94.87
**control1**	40	35	1	1	3	87.50	35	2	2	1	87.50	37	0	1	2	92.50
**control10**	42	42	0	0	0	100	41	0	1	0	97.62	41	0	1	0	97.62
**control11**	25	24	0	0	1	96.00	25	0	0	0	100	25	0	0	0	100
**control12**	44	43	0	0	1	97.73	43	1	0	0	97.73	43	1	0	0	97.73
**control13**	39	38	0	0	1	97.44	38	0	1	0	97.44	39	0	0	0	100
**control14**	34	34	0	0	0	100	33	0	0	1	97.06	34	0	0	0	100
**control15**	43	32	3	8	0	74.42	33	1	7	2	76.74	38	1	4	0	88.37
**control16**	33	30	0	3	0	90.91	32	0	0	1	96.97	32	0	1	0	96.97
**control2**	38	34	0	3	1	89.47	32	1	3	2	84.21	36	1	0	1	94.74
**control3**	43	42	0	1	0	97.67	42	0	0	1	97.67	42	0	0	1	97.67
**control4**	42	41	0	0	1	97.62	39	0	2	1	92.86	41	0	1	0	97.62
**control5**	40	34	0	6	0	85.00	37	0	3	0	92.50	37	0	2	1	92.50
**control6**	40	34	0	6	0	85.00	32	0	7	1	80.00	37	0	2	1	92.50
**control7**	34	32	1	1	0	94.12	34	0	0	0	100	34	0	0	0	100
**control8**	37	31	0	3	3	83.78	33	0	3	1	89.19	33	0	3	1	89.19
**control9**	38	37	0	1	0	97.37	35	0	3	0	92.11	38	0	0	0	100
**hunt1**	53	2	2	49	0	92.45	1	1	49	2	92.45	0	0	53	0	100
**hunt10**	33	0	3	29	1	87.88	0	1	32	0	96.97	1	2	29	1	87.88
**hunt11**	47	0	0	45	2	95.74	0	0	39	8	82.98	2	1	36	8	76.60
**hunt12**	50	5	2	42	1	84.00	2	4	43	1	86.00	3	0	46	1	92.00
**hunt13**	21	1	4	15	1	71.43	1	5	14	1	66.67	1	4	15	1	71.43
**hunt14**	40	1	0	39	0	97.50	1	0	39	0	97.50	0	0	40	0	100
**hunt15**	34	2	1	21	10	61.76	0	0	32	2	94.12	0	2	29	3	85.29

**hunt16**	37	1	4	31	1	83.78	1	3	31	2	83.78	1	3	30	3	81.08
**hunt17**	44	6	2	35	1	79.55	1	0	43	0	97.73	4	2	37	1	84.09
**hunt18**	38	7	3	26	2	68.42	6	4	27	1	71.05	2	1	33	2	86.84
**hunt19**	29	0	1	28	0	96.55	0	0	27	2	93.10	1	1	27	0	93.10
**hunt2**	29	1	1	24	3	82.76	1	1	26	1	89.66	3	0	25	1	86.21
**hunt20**	38	0	1	37	0	97.37	0	1	37	0	97.37	0	0	38	0	100
**hunt3**	31	1	0	29	1	93.55	2	0	26	3	83.87	2	0	27	2	87.10
**hunt4**	35	1	3	30	1	85.71	0	0	35	0	100	4	0	29	2	82.86
**hunt5**	36	0	0	36	0	100	0	1	32	3	88.89	0	1	35	0	97.22
**hunt6**	33	2	1	30	0	90.91	5	0	27	1	81.82	3	2	26	2	78.79
**hunt7**	32	0	2	30	0	93.75	1	0	30	1	93.75	0	1	30	1	93.75
**hunt8**	40	0	0	40	0	100	1	0	39	0	97.50	2	0	38	0	95.00
**hunt9**	27	3	1	22	1	81.48	0	2	25	0	92.59	4	0	23	0	85.19
**park1**	37	1	1	3	32	86.49	1	0	5	31	83.78	4	1	4	28	75.68
**park10**	34	1	0	2	31	91.18	0	1	3	30	88.24	1	0	3	30	88.24
**park11**	33	0	1	1	31	93.94	3	0	1	29	87.88	1	0	3	29	87.88
**park12**	41	1	1	1	38	92.68	5	0	1	35	85.37	0	0	1	40	97.56
**park13**	42	1	0	1	40	95.24	2	0	1	39	92.86	2	0	0	40	95.24
**park14**	40	5	2	5	28	70.00	3	0	10	27	67.50	2	2	4	32	80.00
**park15**	32	0	0	1	31	96.88	0	0	1	31	96.88	1	0	1	30	93.75
**park2**	37	3	0	1	33	89.19	1	0	4	32	86.49	0	0	2	35	94.59
**park3**	43	0	1	2	40	93.02	0	2	1	40	93.02	1	1	1	40	93.02
**park4**	35	0	1	0	34	97.14	1	0	1	33	94.29	1	0	1	33	94.29
**park5**	35	1	0	3	31	88.57	0	0	2	33	94.29	0	0	1	34	97.14
**park6**	41	0	0	6	35	85.37	1	3	6	31	75.61	1	0	3	37	90.24
**park7**	33	0	3	3	27	81.82	0	1	3	29	87.88	0	0	3	30	90.91
**park8**	27	0	2	8	17	62.96	1	1	6	19	70.37	0	2	6	19	70.37
**park9**	43	1	0	1	41	95.35	1	0	2	40	93.02	2	0	0	41	95.35

Table S8. Subject-wise distribution of predicted gait-cycle classes and classification accuracy (%) for the retrained MobileNetV2 model (α = 1.4) on the test subset, using the Trajectory Map, Derivative Map, and Occupancy Heatmap representations. Abbreviations. C: CONTROL (healthy control subjects); A: ALS (amyotrophic lateral sclerosis); H: HUNT (Huntington's disease); P: PARK (Parkinson's disease); n: number of gait cycles; Acc: Accuracy - percentage of correctly classified gait cycles for the corresponding subject; α: MobileNetV2 width multiplier.

GNDD Database		Trajectory Map					Derivative Map					Occupancy Heatmap				
Patient ID	Testset Gait cycles, n	C n	A, n	H, n	P, n	Acc (%)	C, n	A, n	H, n	P, n	Acc (%)	C, n	A, n	H, n	P, n	Acc (%)
als1	26	0	26	0	0	100	0	24	1	1	92.31	0	23	1	2	88.46
als10	36	0	35	0	1	97.22	0	34	2	0	94.44	0	35	1	0	97.22
als11	31	0	31	0	0	100	1	29	1	0	93.55	0	29	0	2	93.55
als12	20	0	19	1	0	95.00	0	19	1	0	95.00	0	20	0	0	100
als13	22	0	22	0	0	100	0	21	1	0	95.45	0	19	1	2	86.36
als2	36	2	33	0	1	91.67	1	35	0	0	97.22	1	35	0	0	97.22
als3	41	0	40	1	0	97.56	0	35	6	0	85.37	0	39	2	0	95.12
als4	21	1	19	1	0	90.48	2	18	1	0	85.71	1	18	2	0	85.71
als5	17	0	16	1	0	94.12	0	14	3	0	82.35	0	17	0	0	100
als6	21	1	19	1	0	90.48	0	18	2	1	85.71	0	19	1	1	90.48
als7	24	0	21	2	1	87.50	0	21	3	0	87.50	0	22	2	0	91.67
als8	33	0	33	0	0	100	1	31	1	0	93.94	0	31	1	1	93.94
als9	39	0	38	1	0	97.44	1	37	1	0	94.87	1	37	1	0	94.87
control1	40	36	1	1	2	90.00	37	0	3	0	92.50	34	0	2	4	85.00
control10	42	40	0	2	0	95.24	40	0	2	0	95.24	42	0	0	0	100
control11	25	25	0	0	0	100	23	1	0	1	92.00	25	0	0	0	100
control12	44	43	0	1	0	97.73	41	0	3	0	93.18	42	0	2	0	95.45
control13	39	39	0	0	0	100	35	0	2	2	89.74	37	0	0	2	94.87
control14	34	34	0	0	0	100	32	0	2	0	94.12	34	0	0	0	100
control15	43	33	1	8	1	76.74	32	2	9	0	74.42	36	1	5	1	83.72
control16	33	33	0	0	0	100	31	0	1	1	93.94	30	0	2	1	90.91
control2	38	35	0	3	0	92.11	30	0	7	1	78.95	38	0	0	0	100
control3	43	38	0	1	4	88.37	40	0	0	3	93.02	39	0	3	1	90.70
control4	42	39	1	0	2	92.86	38	0	4	0	90.48	40	0	1	1	95.24
control5	40	32	2	4	2	80.00	34	4	1	1	85.00	34	1	3	2	85.00
control6	40	34	0	4	2	85.00	32	0	7	1	80.00	35	0	4	1	87.50
control7	34	31	0	3	0	91.18	32	0	2	0	94.12	33	0	1	0	97.06
control8	37	34	0	2	1	91.89	31	0	5	1	83.78	35	0	2	0	94.59
control9	38	36	1	1	0	94.74	35	0	3	0	92.11	36	0	1	1	94.74
hunt1	53	2	2	49	0	92.45	2	2	48	1	90.57	0	0	52	1	98.11
hunt10	33	0	5	28	0	84.85	0	1	32	0	96.97	0	3	30	0	90.91
hunt11	47	2	2	35	8	74.47	0	1	43	3	91.49	0	1	44	2	93.62
hunt12	50	1	4	43	2	86.00	0	3	46	1	92.00	1	1	47	1	94.00
hunt13	21	0	8	10	3	47.62	1	5	13	2	61.90	1	4	13	3	61.90
hunt14	40	1	0	39	0	97.50	0	0	40	0	100	0	0	39	1	97.50
hunt15	34	0	7	16	11	47.06	0	0	34	0	100	0	0	29	5	85.29

**hunt16**	37	2	5	28	2	75.68	0	2	35	0	94.59	2	2	30	3	81.08
**hunt17**	44	4	1	39	0	88.64	0	0	44	0	100	1	1	42	0	95.45
**hunt18**	38	3	3	28	4	73.68	1	3	33	1	86.84	1	2	34	1	89.47
**hunt19**	29	2	3	24	0	82.76	2	0	23	4	79.31	0	1	28	0	96.55
**hunt2**	29	0	0	29	0	100	1	0	27	1	93.10	0	2	25	2	86.21
**hunt20**	38	0	0	38	0	100	0	0	38	0	100	1	0	37	0	97.37
**hunt3**	31	2	1	25	3	80.65	0	0	30	1	96.77	0	0	30	1	96.77
**hunt4**	35	2	2	30	1	85.71	1	0	33	1	94.29	1	0	32	2	91.43
**hunt5**	36	1	1	34	0	94.44	0	0	35	1	97.22	1	0	34	1	94.44
**hunt6**	33	3	2	28	0	84.85	0	0	31	2	93.94	1	0	29	3	87.88
**hunt7**	32	0	2	29	1	90.63	0	1	31	0	96.88	0	0	32	0	100
**hunt8**	40	0	0	40	0	100	1	0	39	0	97.50	0	0	39	1	97.50
**hunt9**	27	1	3	23	0	85.19	0	2	25	0	92.59	2	0	25	0	92.59
**park1**	37	1	1	3	32	86.49	1	1	5	30	81.08	3	0	1	33	89.19
**park10**	34	0	1	1	32	94.12	0	2	2	30	88.24	0	1	1	32	94.12
**park11**	33	0	0	1	32	96.97	0	0	4	29	87.88	0	0	1	32	96.97
**park12**	41	2	0	1	38	92.68	1	1	1	38	92.68	0	0	0	41	100
**park13**	42	2	0	1	39	92.86	2	0	1	39	92.86	2	0	0	40	95.24
**park14**	40	5	1	2	32	80.00	1	2	7	30	75.00	3	0	11	26	65.00
**park15**	32	0	1	0	31	96.88	0	0	0	32	100	0	0	0	32	100
**park2**	37	1	0	0	36	97.30	1	0	5	31	83.78	1	0	3	33	89.19
**park3**	43	0	2	2	39	90.70	0	1	1	41	95.35	0	0	2	41	95.35
**park4**	35	0	2	0	33	94.29	0	1	1	33	94.29	0	0	0	35	100
**park5**	35	0	0	3	32	91.43	0	1	3	31	88.57	0	0	0	35	100
**park6**	41	0	2	2	37	90.24	0	1	7	33	80.49	0	0	4	37	90.24
**park7**	33	2	2	0	29	87.88	0	1	2	30	90.91	1	0	1	31	93.94
**park8**	27	0	2	6	19	70.37	0	1	12	14	51.85	0	0	9	18	66.67
**park9**	43	2	1	0	40	93.02	2	1	0	40	93.02	0	0	2	41	95.35

Table S9. Subject-wise distribution of predicted gait-cycle classes and classification accuracy (%) for the fine-tuned MobileNetV2 model (α = 0.35) on the test subset, using the Trajectory Map, Derivative Map, and Occupancy Heatmap representation. Abbreviations. C: CONTROL (healthy control subjects); A: ALS (amyotrophic lateral sclerosis); H: HUNT (Huntington's disease); P: PARK (Parkinson's disease); n: number of gait cycles; Acc: Accuracy - percentage of correctly classified gait cycles for the corresponding subject; α: MobileNetV2 width multiplier.

GNDD Database		Trajectory Map					Derivative Map					Occupancy Heatmap				
Patient ID	Testset Gait cycles, n	C n	A, n	H, n	P, n	Acc (%)	C, n	A, n	H, n	P, n	Acc (%)	C, n	A, n	H, n	P, n	Acc (%)
**als1**	26	0	26	0	0	100	0	26	0	0	100	0	26	0	0	100
**als10**	36	0	35	1	0	97.22	0	35	1	0	97.22	0	35	1	0	97.22
**als11**	31	0	30	1	0	96.77	0	30	1	0	96.77	0	29	2	0	93.55
**als12**	20	0	20	0	0	100	0	19	1	0	95.00	0	19	1	0	95.00
**als13**	22	0	21	1	0	95.45	0	22	0	0	100	0	20	1	1	90.91
**als2**	36	1	34	1	0	94.44	0	34	0	2	94.44	1	35	0	0	97.22
**als3**	41	0	39	2	0	95.12	0	40	1	0	97.56	0	40	0	1	97.56
**als4**	21	0	17	2	2	80.95	1	18	0	2	85.71	0	18	1	2	85.71
**als5**	17	0	16	1	0	94.12	0	14	3	0	82.35	0	16	1	0	94.12
**als6**	21	0	21	0	0	100	0	19	2	0	90.48	0	20	0	1	95.24
**als7**	24	0	21	2	1	87.50	0	23	1	0	95.83	0	20	2	2	83.33
**als8**	33	0	31	2	0	93.94	0	32	1	0	96.97	0	31	2	0	93.94
**als9**	39	0	37	1	1	94.87	0	38	1	0	97.44	0	37	2	0	94.87
**control1**	40	36	0	2	2	90.00	37	1	1	1	92.50	38	0	1	1	95.00
**control10**	42	42	0	0	0	100	42	0	0	0	100	41	0	1	0	97.62
**control11**	25	25	0	0	0	100	25	0	0	0	100	25	0	0	0	100
**control12**	44	43	0	0	1	97.73	43	0	0	1	97.73	43	1	0	0	97.73
**control13**	39	39	0	0	0	100	38	0	1	0	97.44	39	0	0	0	100
**control14**	34	34	0	0	0	100	34	0	0	0	100	33	0	1	0	97.06
**control15**	43	38	1	4	0	88.37	41	0	2	0	95.35	39	1	2	1	90.70
**control16**	33	32	0	1	0	96.97	33	0	0	0	100	33	0	0	0	100
**control2**	38	36	0	1	1	94.74	36	0	0	2	94.74	35	0	3	0	92.11
**control3**	43	42	0	1	0	97.67	42	0	0	1	97.67	42	0	1	0	97.67
**control4**	42	41	0	1	0	97.62	41	0	0	1	97.62	41	0	1	0	97.62
**control5**	40	38	0	2	0	95.00	38	2	0	0	95.00	36	0	3	1	90.00
**control6**	40	37	0	3	0	92.50	35	0	4	1	87.50	40	0	0	0	100
**control7**	34	33	0	1	0	97.06	34	0	0	0	100	34	0	0	0	100
**control8**	37	34	0	1	2	91.89	34	0	2	1	91.89	34	0	2	1	91.89
**control9**	38	38	0	0	0	100	37	0	1	0	97.37	38	0	0	0	100
**hunt1**	53	0	1	52	0	98.11	0	1	50	2	94.34	1	0	52	0	98.11
**hunt10**	33	0	0	32	1	96.97	0	1	32	0	96.97	0	0	32	1	96.97
**hunt11**	47	1	0	45	1	95.74	0	0	46	1	97.87	1	0	42	4	89.36
**hunt12**	50	0	0	50	0	100	0	0	46	4	92.00	0	0	50	0	100
**hunt13**	21	1	1	19	0	90.48	1	3	17	0	80.95	0	1	19	1	90.48
**hunt14**	40	0	0	40	0	100	1	0	39	0	97.50	0	0	40	0	100
**hunt15**	34	0	0	26	8	76.47	1	0	32	1	94.12	0	0	33	1	97.06

**hunt16**	37	1	1	33	2	89.19	0	1	34	2	91.89	0	2	33	2	89.19
**hunt17**	44	1	0	43	0	97.73	2	0	41	1	93.18	2	1	41	0	93.18
**hunt18**	38	4	1	33	0	86.84	5	1	31	1	81.58	0	0	36	2	94.74
**hunt19**	29	1	1	27	0	93.10	0	1	27	1	93.10	0	1	28	0	96.55
**hunt2**	29	1	0	25	3	86.21	0	0	28	1	96.55	0	0	28	1	96.55
**hunt20**	38	0	0	38	0	100	0	0	38	0	100	0	0	38	0	100
**hunt3**	31	0	0	31	0	100	1	0	29	1	93.55	0	0	31	0	100
**hunt4**	35	0	0	33	2	94.29	0	0	35	0	100	0	0	35	0	100
**hunt5**	36	0	0	36	0	100	0	0	35	1	97.22	0	0	36	0	100
**hunt6**	33	0	1	32	0	96.97	2	0	30	1	90.91	0	2	30	1	90.91
**hunt7**	32	0	0	32	0	100	0	0	32	0	100	0	0	31	1	96.88
**hunt8**	40	1	0	39	0	97.50	0	0	40	0	100	1	0	39	0	97.50
**hunt9**	27	0	0	26	1	96.30	0	1	26	0	96.30	2	1	24	0	88.89
**park1**	37	0	0	2	35	94.59	1	0	4	32	86.49	1	1	1	34	91.89
**park10**	34	0	0	1	33	97.06	0	0	2	32	94.12	0	0	2	32	94.12
**park11**	33	0	0	1	32	96.97	0	0	1	32	96.97	0	0	2	31	93.94
**park12**	41	1	1	0	39	95.12	2	0	0	39	95.12	0	0	0	41	100
**park13**	42	0	0	1	41	97.62	1	0	0	41	97.62	1	0	1	40	95.24
**park14**	40	2	0	6	32	80.00	1	0	4	35	87.50	0	3	3	34	85.00
**park15**	32	0	0	1	31	96.88	0	0	0	32	100	0	0	1	31	96.88
**park2**	37	0	0	1	36	97.30	0	0	0	37	100	1	0	0	36	97.30
**park3**	43	0	0	1	42	97.67	0	0	2	41	95.35	0	0	1	42	97.67
**park4**	35	0	1	0	34	97.14	1	0	0	34	97.14	0	0	0	35	100
**park5**	35	0	0	1	34	97.14	0	0	1	34	97.14	0	0	0	35	100
**park6**	41	0	0	2	39	95.12	0	0	1	40	97.56	0	0	2	39	95.12
**park7**	33	0	2	1	30	90.91	0	1	2	30	90.91	0	1	2	30	90.91
**park8**	27	0	0	6	21	77.78	1	0	3	23	85.19	0	0	3	24	88.89
**park9**	43	2	0	0	41	95.35	1	0	0	42	97.67	0	0	1	42	97.67

https://doi.org/10.3390/xxxxx

Table S10. Subject-wise distribution of predicted gait-cycle classes and classification accuracy (%) for the fine-tuned MobileNetV2 model (α = 1.4) on the test subset, using the Trajectory Map, Derivative Map, and Occupancy Heatmap representations. Abbreviations. C: CONTROL (healthy control subjects); A: ALS (amyotrophic lateral sclerosis); H: HUNT (Huntington's disease); P: PARK (Parkinson's disease); n: number of gait cycles; Acc: Accuracy - percentage of correctly classified gait cycles for the corresponding subject; α: MobileNetV2 width multiplier.

GNDD Database		Trajectory Map					Derivative Map					Occupancy Heatmap				
**Patient ID**	**Testset Gait cycles, n**	**C n**	**A, n**	**H, n**	**P, n**	**Acc (%)**	**C, n**	**A, n**	**H, n**	**P, n**	**Acc (%)**	**C, n**	**A, n**	**H, n**	**P, n**	**Acc (%)**
**als1**	26	0	26	0	0	100	0	26	0	0	100	0	26	0	0	100
**als10**	36	0	35	1	0	97.22	0	35	1	0	97.22	0	35	1	0	97.22
**als11**	31	0	31	0	0	100	0	30	1	0	96.77	0	30	0	1	96.77
**als12**	20	0	19	1	0	95.00	0	20	0	0	100	0	20	0	0	100
**als13**	22	0	22	0	0	100	1	21	0	0	95.45	0	22	0	0	100
**als2**	36	2	34	0	0	94.44	1	34	1	0	94.44	1	33	2	0	91.67
**als3**	41	0	38	3	0	92.68	0	39	2	0	95.12	1	40	0	0	97.56
**als4**	21	0	20	1	0	95.24	2	18	0	1	85.71	0	20	1	0	95.24
**als5**	17	0	16	1	0	94.12	0	15	2	0	88.24	0	15	2	0	88.24
**als6**	21	0	21	0	0	100	0	19	2	0	90.48	0	20	0	1	95.24
**als7**	24	0	23	1	0	95.83	0	24	0	0	100	0	23	1	0	95.83
**als8**	33	0	32	1	0	96.97	0	33	0	0	100	0	32	1	0	96.97
**als9**	39	0	38	1	0	97.44	1	37	1	0	94.87	0	37	2	0	94.87
**control1**	40	37	0	2	1	92.50	37	0	1	2	92.50	37	0	1	2	92.50
**control10**	42	42	0	0	0	100	42	0	0	0	100	42	0	0	0	100
**control11**	25	25	0	0	0	100	25	0	0	0	100	25	0	0	0	100
**control12**	44	44	0	0	0	100	43	0	1	0	97.73	43	0	1	0	97.73
**control13**	39	38	0	0	1	97.44	39	0	0	0	100	39	0	0	0	100
**control14**	34	34	0	0	0	100	34	0	0	0	100	34	0	0	0	100
**control15**	43	38	1	3	1	88.37	38	0	5	0	88.37	39	0	4	0	90.70
**control16**	33	33	0	0	0	100	33	0	0	0	100	33	0	0	0	100
**control2**	38	36	0	2	0	94.74	33	0	5	0	86.84	37	0	0	1	97.37
**control3**	43	42	0	0	1	97.67	42	0	0	1	97.67	42	0	0	1	97.67
**control4**	42	41	1	0	0	97.62	41	0	1	0	97.62	41	0	1	0	97.62
**control5**	40	39	0	1	0	97.50	37	2	0	1	92.50	38	0	2	0	95.00
**control6**	40	38	0	1	1	95.00	36	0	3	1	90.00	38	0	1	1	95.00
**control7**	34	32	0	2	0	94.12	32	0	2	0	94.12	33	0	1	0	97.06
**control8**	37	35	0	1	1	94.59	34	0	3	0	91.89	35	0	2	0	94.59
**control9**	38	38	0	0	0	100	38	0	0	0	100	38	0	0	0	100
**hunt1**	53	0	1	52	0	98.11	0	1	51	1	96.23	0	1	52	0	98.11
**hunt10**	33	0	1	32	0	96.97	0	1	31	1	93.94	0	2	31	0	93.94
**hunt11**	47	1	0	43	3	91.49	0	1	42	4	89.36	0	1	45	1	95.74
**hunt12**	50	0	1	49	0	98.00	1	2	46	1	92.00	0	0	50	0	100
**hunt13**	21	0	2	18	1	85.71	1	3	17	0	80.95	2	1	16	2	76.19
**hunt14**	40	1	0	39	0	97.50	1	0	39	0	97.50	0	0	40	0	100
**hunt15**	34	0	0	28	6	82.35	0	0	34	0	100	0	0	30	4	88.24

https://doi.org/10.3390/xxxxx

**hunt16**	37	0	0	35	2	94.59	0	1	36	0	97.30	0	1	36	0	97.30
**hunt17**	44	1	0	43	0	97.73	2	0	42	0	95.45	1	0	43	0	97.73
**hunt18**	38	1	0	34	3	89.47	2	0	36	0	94.74	2	0	35	1	92.11
**hunt19**	29	0	0	29	0	100	0	0	27	2	93.10	0	0	29	0	100
**hunt2**	29	0	0	29	0	100	2	0	27	0	93.10	1	1	27	0	93.10
**hunt20**	38	0	0	38	0	100	0	0	38	0	100	0	0	38	0	100
**hunt3**	31	1	0	29	1	93.55	0	0	30	1	96.77	1	0	30	0	96.77
**hunt4**	35	0	0	34	1	97.14	0	0	35	0	100	0	0	35	0	100
**hunt5**	36	0	2	33	1	91.67	0	0	36	0	100	0	0	36	0	100
**hunt6**	33	1	1	31	0	93.94	2	0	31	0	93.94	0	0	33	0	100
**hunt7**	32	0	0	31	1	96.88	0	0	32	0	100	0	0	32	0	100
**hunt8**	40	0	0	40	0	100	0	0	40	0	100	1	0	39	0	97.50
**hunt9**	27	0	1	25	1	92.59	1	1	25	0	92.59	1	0	26	0	96.30
**park1**	37	0	1	0	36	97.30	1	1	2	33	89.19	2	0	2	33	89.19
**park10**	34	0	0	0	34	100	1	0	1	32	94.12	0	1	1	32	94.12
**park11**	33	0	0	0	33	100	0	0	1	32	96.97	0	0	0	33	100
**park12**	41	0	0	0	41	100	3	0	0	38	92.68	0	0	1	40	97.56
**park13**	42	0	0	2	40	95.24	0	0	1	41	97.62	1	0	0	41	97.62
**park14**	40	0	0	4	36	90.00	0	0	3	37	92.50	2	0	4	34	85.00
**park15**	32	0	0	0	32	100	0	0	0	32	100	0	0	0	32	100
**park2**	37	0	0	0	37	100	1	0	0	36	97.30	0	0	1	36	97.30
**park3**	43	0	0	1	42	97.67	0	1	1	41	95.35	1	0	1	41	95.35
**park4**	35	0	1	0	34	97.14	0	1	1	33	94.29	0	0	0	35	100
**park5**	35	0	0	0	35	100	0	0	0	35	100	0	0	0	35	100
**park6**	41	0	0	1	40	97.56	0	0	2	39	95.12	0	1	0	40	97.56
**park7**	33	1	0	1	31	93.94	0	1	1	31	93.94	0	1	1	31	93.94
**park8**	27	0	0	5	22	81.48	0	0	4	23	85.19	0	0	3	24	88.89
**park9**	43	2	0	0	41	95.35	0	0	1	42	97.67	0	0	2	41	95.35

Disclaimer/Publisher's Note: The statements, opinions and data contained in all publications are solely those of the individual author(s) and contributor(s) and not of MDPI and/or the editor(s). MDPI and/or the editor(s) disclaim responsibility for any injury to people or property resulting from any ideas, methods, instructions or products referred to in the content.
